# Possible cases of leprosy from the Late Copper Age (3780-3650 cal BC) in Hungary

**DOI:** 10.1371/journal.pone.0185966

**Published:** 2017-10-12

**Authors:** Kitti Köhler, Antónia Marcsik, Péter Zádori, Gergely Biro, Tamás Szeniczey, Szilvia Fábián, Gábor Serlegi, Tibor Marton, Helen D. Donoghue, Tamás Hajdu

**Affiliations:** 1 Institute of Archaeology, Research Centre for the Humanities, Hungarian Academy of Sciences, Budapest, Hungary; 2 Retired associate professor, University of Szeged, Szeged, Hungary; 3 Health Centre, Kaposvár University, Kaposvár, Hungary; 4 Department of Biological Anthropology, Institute of Biology, Faculty of Science, Eötvös Loránd University, Budapest, Hungary; 5 Department of Archaeological Excavations and Artefact Processing, Hungarian National Museum, Budapest, Hungary; 6 Centre for Clinical Microbiology, Royal Free Campus, University College London, London, United Kingdom; Hebrew University, ISRAEL

## Abstract

At the Abony-Turjányos dűlő site, located in Central Hungary, a rescue excavation was carried out. More than 400 features were excavated and dated to the Protoboleráz horizon, at the beginning of the Late Copper Age in the Carpathian Basin, between 3780–3650 cal BC. Besides the domestic and economic units, there were two special areas, with nine-nine pits that differed from the other archaeological features of the site. In the northern pit group seven pits contained human remains belonging to 48 individuals. Some of them were buried carefully, while others were thrown into the pits. The aim of this study is to present the results of the paleopathological and molecular analysis of human remains from this Late Copper Age site. The ratio of neonates to adults was high, 33.3%. Examination of the skeletons revealed a large number of pathological cases, enabling reconstruction of the health profile of the buried individuals. Based on the appearance and frequency of healed ante- and peri mortem trauma, inter-personal (intra-group) violence was characteristic in the Abony Late Copper Age population. However other traces of paleopathology were observed on the bones that appear not to have been caused by warfare or inter-group violence. The remains of one individual demonstrated a rare set of bone lesions that indicate the possible presence of leprosy (Hansen’s disease). The most characteristic lesions occurred on the bones of the face, including erosion of the nasal aperture, atrophy of the anterior nasal spine, inflammation of the nasal bone and porosity on both the maxilla and the bones of the lower legs. In a further four cases, leprosy infection is suspected but other infections cannot be excluded. The morphologically diagnosed possible leprosy case significantly modifies our knowledge about the timescale and geographic spread of this specific infectious disease. However, it is not possible to determine the potential connections between the cases of possible leprosy and the special burial circumstances.

## Introduction

### Archaeological background

**Fig 1:**
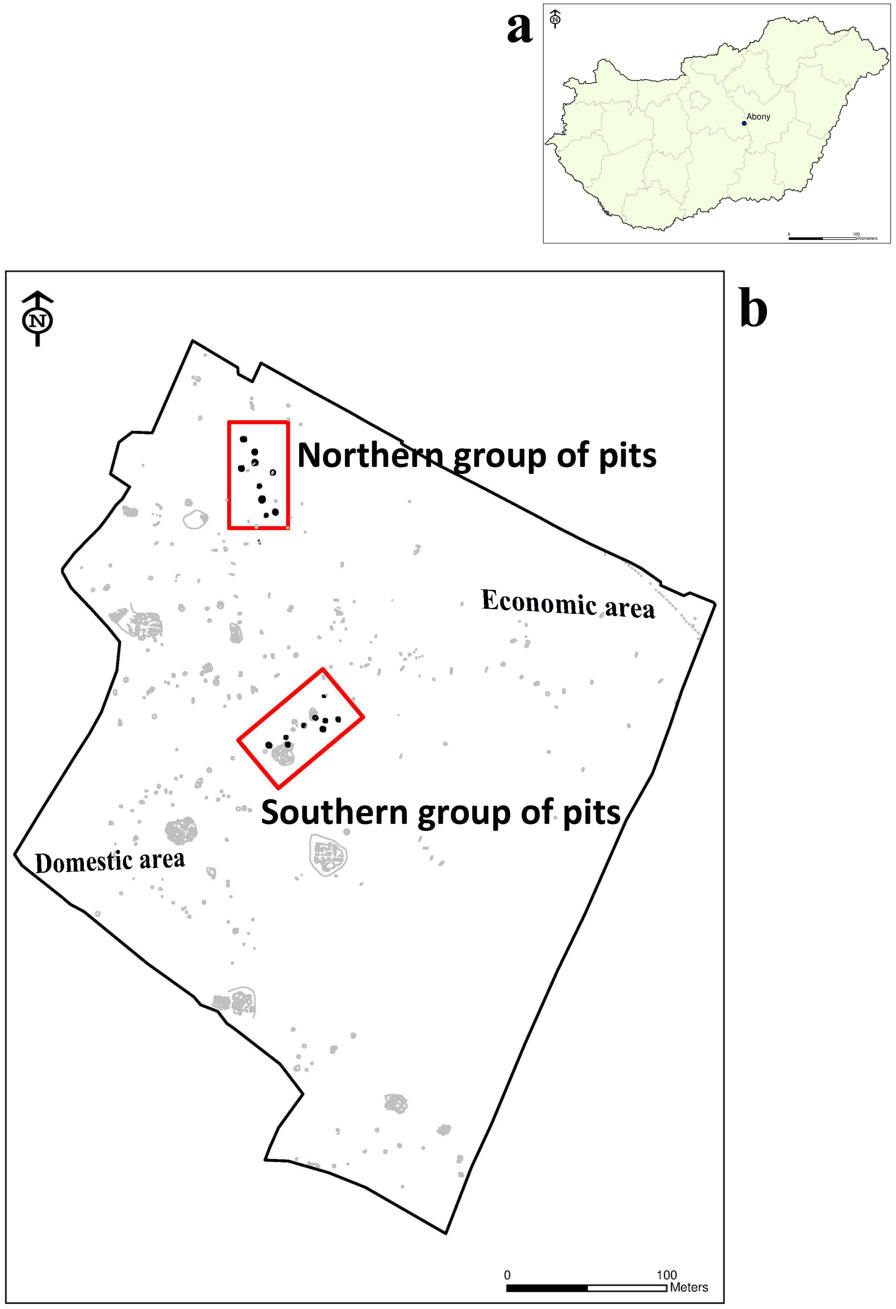
a: Location of Abony-Turjányos dűlő site. b: Site map of Abony-Turjányos dűlő.

The Abony-Turjányos dűlő site (Fig 1a) is located in central Hungary, where a rescue excavation was carried out between 2004 and 2008. In the site more than 400 features were excavated and dated to the ProtoBoleráz horizon, immediately before the Baden cultural complex of the Late Copper Age in the Carpathian Basin [1–5]. The Accelerator Mass Spectroscopy (AMS) radiocarbon dating indicates that the settlement was used between 3780 and 3650 cal BC. The measurements was made at the Vienna Environmental Research Accelerator (VERA) on samples of animal and human bones [5].

In the southern and southwestern part of the site different kinds of pits—such as storage pits, refuse pits and pit complexes—were unearthed that contained large numbers of collapsed vessels (Fig 1b). The pits also yielded raw materials for housing and a great amount of daub rubble. Some of the daubs were found with imprints of wooden stick and boards, which seem to have belonged to the structure of former houses with wattle and daub walls. These remains indicated that this were the domestic part of the settlement. The eastern and northeastern part of the site was the economic area of the settlement. The excavators assumed that the elongated pits excavated here may have served some function other than storage or clay extraction so could have been used in a special work process [5].

In the economic unit of the settlement there were two special (a northern and southern) area, each with pits that were different in all aspects from the other archaeological features of the site. In the northern group seven pits contained human remains belonging to 48 individuals. Some of them were buried carefully, while others had been thrown into the pits. The skeletons were in clearly separated layers. The pits contained black humus soil and also sterile loess layers. In the rest of these sacrificial pits (bothroi), beside the human burials, animal bones, intact ceramic vessels and sherds were deposited in layers, which suggest the regular repetition of burial activities. The southern group with nine pits was recovered later, but contained no human remains [5].

Other mass graves or multiple burials dating from the Late Copper Age have been found within settlements from the Carpathian Basin, including: Palotabozsok [6,7] Tikos-Homokgödrök [8], Balatonszemes-Szemesi Berek [6,7,9], Balatonőszöd-Temetői dűlő [7,10], Balatonboglár [7,11] and Kaposújlak-Várdomb [7,12]. In the archaeological literature these pits are usually described as sacrificial burials, ritual murders or it is suggested they were connected to warfare or to an epidemic that resulted in the death of several individuals almost simultaneously [6,7,13]. The origin of the Late Copper Age Abony and the other few known contemporary adjacent populations are almost unknown, due to the lack of comparative physical anthropological, ancient DNA and stable isotope data about earlier and contemporary human groups.

During the past two decades, archaeological activity in Hungary has furnished a large number of new cases with paleopathological markers. At the same time, the intensive study of these specific infectious diseases and the development of molecular diagnostic methods has increased the number of publications in this field [14–24].

The aim of this study is to present the results of the paleopathological and molecular analysis of human remains from the Abony Late Copper Age site.

## Methods

### Paleopathological analysis

The estimation of age-at-death and the sex determination were based on the methods commonly used in physical anthropology [25–32]. The paleopathological lesions were investigated macroscopically [33–35]. Due to the possible ritual function of the pits, there was particular emphasis on any paleopathological alterations that might indicate inter- or intragroup violence, or the presence of infections, such as endocranial lesions, periosteal appositions and porotic hyperostosis [36–40]. Special attention was paid to porotic hyperostosis that can be the sign of a weakened immune system. However, hematological disorders can also indicate an increased susceptibility to infections [41]. Porotic hyperostosis was assessed using a 4-grade-scale: no bone lesion; porotic, cribrotic and trabecular forms [42].

The analysis was supplemented by the investigation of possible signs of ante- and peri- mortem trauma, which can indicate the lifestyle and may also shed light on the circumstances of the death of these individuals. In the trauma analysis, bones were analysed according to Lovell [43], Ortner [35] and Rodríguez-Martín [44].

Computed tomography (CT) examinations were performed using a Siemens Somatom Definition Flash DSCT 2x128 scanner (Siemens AG, Erlangen, Germany). Primary images were obtained using a slice thickness of 0.4 mm. Secondary multiplanar and volume rendering technique (VRT) reconstructions were performed for better visualisation.

### Molecular examination for *Mycobacterium leprae aDNA*

#### DNA extraction

The recommended protocols of ancient DNA (aDNA) work [45] were followed, with separate rooms for different stages of the process. Bone scrapings were taken from the following four specimens: feature 257 S20 –inferior nasal concha; feature 263 S25 –hard palate; feature 263 S29 –maxillary alveolar process; and feature 263 S36 –inferior nasal spine. Scrapings were crushed in a sterile pestle and mortar. Next, 50–70 mg of each sample was transferred into a 2ml screw-capped Eppendorf tube containing demineralisation solution (EDTA/Proteinase K). Tubes were incubated in a 56°C heating block and mixed daily for 3 days when the samples were solubilised [15]. Samples were then divided and one aliquot treated with 40μl of 0.1 mol^−1^ of N-phenacylthiozolium bromide (PTB), to cleave any covalent cross-links thus enabling DNA strand separation and amplification [46]. To complete disaggregation of the samples the 2ml tube contents were transferred into 9ml tubes of NucliSens^®^ (bioMérieux) lysis buffer containing 5 mol^-1^ guanidium thiocyanate solution, and incubated at 56°C. Negative extraction controls were processed in parallel with sample tubes.

Tubes were placed on a rotator for 4 hours, snap-frozen in liquid nitrogen and thawed in a 65°C waterbath. This was repeated twice. Sample were spun at 5000g for 15 mins at 5°C and the supernatants carefully removed into clean, sterile tubes. To capture DNA the supernatants were mixed with 50μl silica suspension (NucliSens^®^) and centrifuged. The silica pellets were washed once with wash buffer (NucliSens^®^), twice with 70% (v/v) ethanol (-20°C) and once with acetone (-20°C). After drying in a heating block, DNA was eluted from the silica using 60μl elution buffer (NucliSens^®^), aliquoted and used immediately or stored at -20°C. Silica supernates (500μl) from PTB-negative samples were also collected from the 9ml tubes of lysis buffer, and the 2.0 ml screw-capped Eppendorf tubes used to wash the silica. After chilling at 5°C, supernates were mixed vigorously for 20 s with 200μl of Protein Precipitation Solution (SLS Ltd., UK) and centrifuged for 3min at 10,000g. Any pellet was discarded and 600μl isopropanol (−20°C) added to 550μl of each supernate. Tubes were mixed by inversion 50 times and spun 3min. Supernates were discarded and tubes washed once with 500μl 70% ethanol (−20°C). After draining, tubes were dried in a heating block. Any precipitated DNA was re-hydrated with 60μl elution buffer (NucliSens^®^), aliquoted and used immediately or stored at −20°C. Negative extraction controls were processed in parallel with the test samples.

Two repeat batches of DNA extractions were performed subsequently, using 53–65 mg and 45–55 mg of crushed scrapings as described above.

#### DNA amplification and detection

Initially *M*. *leprae* DNA was sought by targeting a specific region of the repetitive element RLEP (37 copies/cell). A two-tube nested PCR was used which give an outer product of 129 base pairs (bp) and a nested PCR product of 99 bp [47]. A hot-start *Taq* polymerase was used to minimise non-specific primer and template binding. Negative DNA extraction and PCR controls were processed alongside the test sample. Detection of PCR product was by agarose gel electrophoresis, stained with ethidium bromide and visualised under ultraviolet light. As aDNA is normally fragmented, a further pair of RLEP primers with a target region of 111bp was also used in a single-stage PCR reaction [48]. As there are reports of leprosy co-infections with tuberculosis, the first batch of DNA extracts were also examined for the presence of *M*. *tuberculosis* aDNA using IS*6110* primers P1 and P2 followed by a nested reaction with primers IS3 and IS4, with target sequences of 123 bp and 92 bp respectively [15]. Subsequently a second specific region of the repetitive element RepLep (15 copies/cell) was also examined, and specific primers and probes were used to enable shorter PCR fragments to be detected in a real-time PCR reactions using both the RLEP and RepLep target regions (S1 Table). The PCR probe mix included 2mM bovine serum albumin to reduce PCR inhibition [49,50], 2.0mM MgCl_2_ and annealing was at 60°C. A hot-start *Taq* polymerase was used to minimize non-specific primer and template binding. Negative DNA extraction and PCR controls were processed alongside the test sample. Amplification was performed in a final volume of 25μl using a RotorGene 3000 (Qiagen) real-time platform [51].

## Material

During the physical anthropological work the analysed human remains were housed in the Institute of Archaeology, Research Centre for the Humanities and later in the Hungarian National Museum. After the archaeological, anthropological and molecular examinations the material will be stored in the Ferenczy Museum Center. The feature and stratigraphic unit numbers of the investigated skeletons are in the S2 Table.

## Results

### Age and sex distribution

During the physical anthropological work we investigated the remains of 48 individuals (Table 1, S2 Table). In the nine pits differences were observed in the number of buried or thrown-in individuals. In two pits there were no human remains (features 248 and 255), while in feature 263 the skeletal remains of 23 individuals were found. In features 247, 249 and 253 there were only fetuses and neonates. There were inhumations of a female and three neonates in feature 251 (S1 Video) and of one male and one child in feature 250. Most of the individuals were buried in feature 257 (9 skeletons—two females, two males, four subadults and one undeterminable adult—S2 Video) and feature 263 (23 skeletons– 10 females, 7 males, 6 subadults).

**Table 1:** The sex and age distribution of the buried individuals.

Age groups	Sex			
	Not known	Males	Females	Total
Fetus, Neonate, 0–1 years-old	16	–	–	16
Infans I. (1–7 years-old)	6	–	–	6
Infans II. (8–14 years-old)	2	–	–	2
Juvenile (15–23 years-old)	–	1	–	1
Adult (24–39 years-old)	1	5	9	15
Mature (40–59 years-old)	–	4	4	8
Senile (60+ years-old)	–	–	–	0
Total	25	10	13	48

Regarding age distribution, the ratio of children, especially with an-age-at death of 0–1 years was high (33.3%). Furthermore, the proportions of juveniles and young adults were low (Table 1).

### Ante and peri-mortem trauma

In our previous study we published the preliminary observations of traumatic lesions in the Abony population [52]. In this recent paper we provided a more detailed analysis of trauma on the human bones of 48 individuals (25 subadults and 23 adults). The results are described in S1 Text, S3 and S4 Tables, S1–S9 Figs.

### Endocranial lesions

Of all the paleopathology that could be detected, there was only one adult case of endocranial lesions (on a left parietal bone of a female), while these were much more frequent amongst children (Table 2). These usually affected several cranial bones in one individual, mainly the parietals and the occipital bone. From the 18 subadult skulls, endocranial lesions were detected in five cases.

**Table 2:** The occurence of endocranial lesions.

Bone	Sub-adults					Adults					Total				
	N_1_	%	N_2_	%	N_1+2_	N_1_	%	N_2_	%	N_1+2_	N_1_	%	N_2_	%	N_1+2_
Frontal	2	11.1	16	88.9	18	0	0.0	23	100.0	23	2	4.4	43	96.6	45
Left parietal	0	0.0	18	100.0	18	1	3.6	22	96.4	23	1	2.2	45	97.8	46
Right parietal	1	5.9	16	94.1	17	0	0.0	23	100.0	23	1	2.4	41	97.6	42
Left temporal	0	0.0	16	100.0	16	0	0.0	23	100.0	23	0	0.0	40	100.0	40
Right temporal	0	0.0	16	100.0	16	0	0.0	23	100.0	23	0	0.0	41	100.0	41
Occipital	5	38.5	8	61.5	13	0	0.0	23	100.0	23	3	7.9	33	92.1	38

N_1_ = Yes, N_2_ = No, N_1+2_ = Sum of the examinable cases

**Fig 2:**
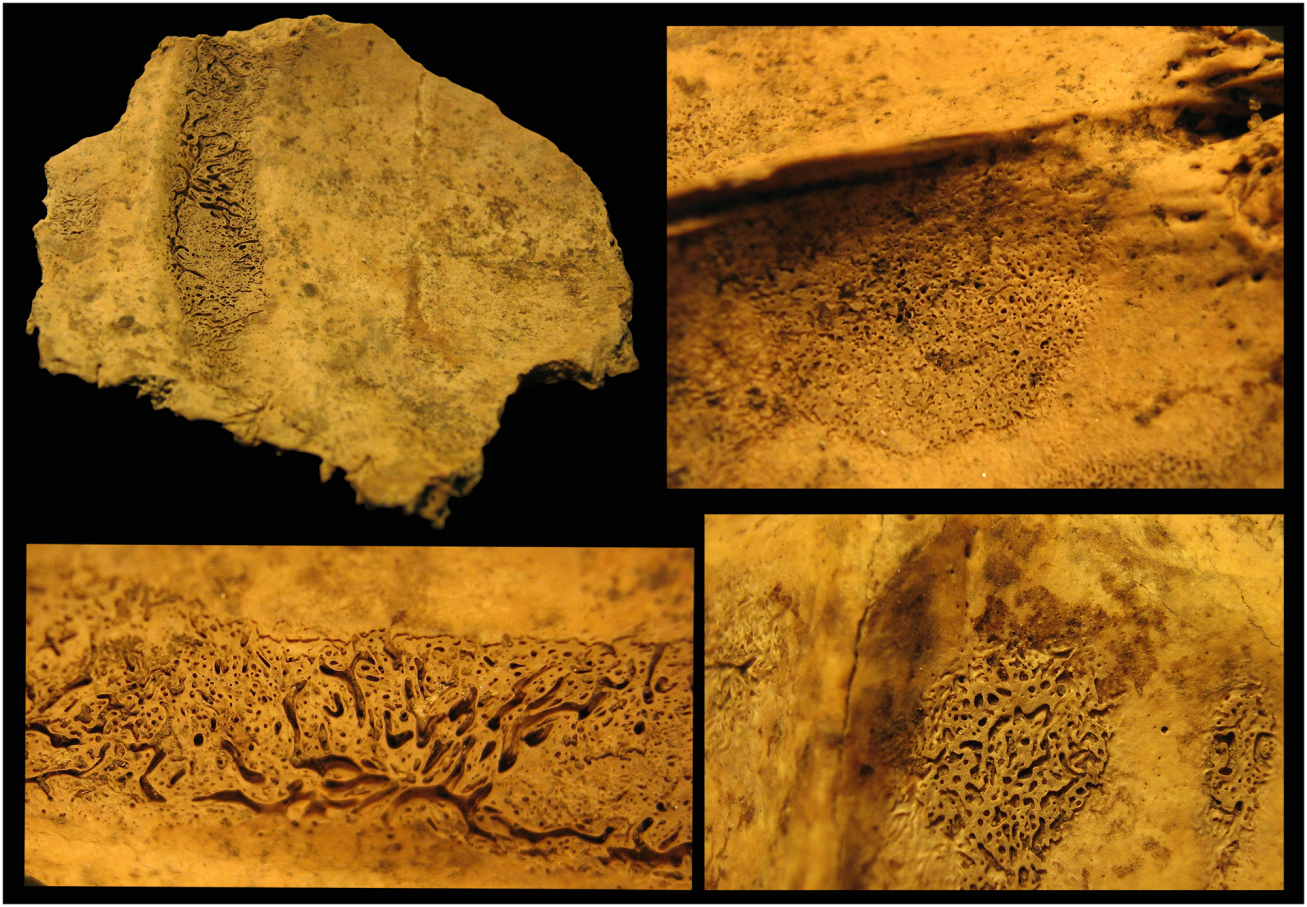
Endocranial patterns on the frontal and occipital bones, feature 263 S38.

In two cases the lesions were present on more bones (feature 257 S17: frontal and occipital bones; feature 263 S38 –Fig 2: frontal, right parietal and occipital bones). In three cases the occipital bones were affected (feature 250 S5; feature 257 S18; feature 263 S27).

### Periosteal lesions

Periosteal lesions can arise from different causes [53,54], but one of the most common is infection [35,55]. Among children periostitis occurs most frequently on the femur and tibia, while among the adults it is mainly on the tibia, the fibula and/or the femur (Table 3). Disaggregated by sex it occured in almost equal proportions, but slightly more frequent among males (Table 4).

**Table 3:** The occurence of periostitis.

	Sub-adults					Adults					Total				
	N_1_	%	N_2_	%	N_1+2_	N_1_	%	N_2_	%	N_1+2_	N_1_	%	N_2_	%	N_1+2_
Left clavicle	1	9.1	10	90.9	11	0	0.0	12	100.0	12	1	4.3	22	95.7	23
Right clavicle	0	0.0	7	100.0	7	1	10.0	9	90.0	10	1	5.9	16	94.1	17
Left humerus	1	5.3	18	94.7	19	1	4.8	20	95.2	21	2	5.0	38	95.0	40
Right humerus	0	0.0	16	100.0	16	1	5.0	19	95.0	20	1	2.8	35	97.2	36
Left radius	0	0.0	9	100.0	9	1	5.3	18	94.7	19	1	3.6	27	96.4	28
Right radius	0	0.0	8	100.0	8	1	5.6	17	94.4	18	1	3.8	25	96.2	26
Left ulna	1	7.7	12	92.3	13	1	5.6	17	94.4	18	2	6.5	29	93.5	31
Right ulna	0	0.0	12	100.0	12	1	5.6	17	94.4	18	1	3.3	29	96.7	30
Left femur	2	13.3	13	86.7	15	5	20.8	19	79.2	24	7	17.9	32	82.1	39
Right femur	2	11.1	16	88.9	18	5	19.2	21	80.8	26	7	15.9	37	84.1	44
Left tibia	2	16.7	10	83.3	12	10	41.7	14	58.3	24	12	33.3	24	66.7	36
Right tibia	5	33.3	10	66.7	15	10	40.0	15	60.0	25	15	37.5	25	62.5	40
Left fibula	0	0.0	8	100.0	8	8	38.1	13	61.9	21	8	27.6	21	72.4	29
Right fibula	0	0.0	7	100.0	7	6	28.6	15	71.4	21	6	21.4	22	78.6	28

N_1_ = Yes, N_2_ = No, N_1+2_ = Sum of the examinable cases

**Table 4:** The occurence of periostitis according to sex.

Bones	Males					Females				
	N_1_	%	N_2_	%	N_1_+N_2_	N_1_	%	N_2_	%	N_1_+N_2_
Left clavicle	0	0.0	2	100.0	2	0	0.0	9	100.0	9
Right clavicle	0	0.0	2	100.0	2	1	12.5	7	87.5	8
Left humerus	1	16.7	5	83.3	6	0	0.0	8	100.0	8
Right humerus	1	16.7	5	83.3	6	0	0.0	9	100.0	9
Left radius	1	16.7	5	83.3	6	0	0.0	9	100.0	9
Right radius	1	16.7	5	83.3	6	0	0.0	8	100.0	8
Left ulna	1	16.7	5	83.3	6	0	0.0	9	100.0	9
Right ulna	1	16.7	5	83.3	6	0	0.0	7	100.0	7
Left femur	2	25.0	6	75.0	8	2	20.0	8	80.0	10
Right femur	2	28.6	5	71.4	7	2	18.2	9	81.8	11
Left tibia	3	42.8	4	57.2	7	5	55.6	4	44.4	9
Right tibia	4	57.2	3	42.8	7	4	44.4	5	55.6	9
Left fibula	4	57.2	3	42.8	7	3	33.3	6	66.7	9
Right fibula	3	50.0	3	50.0	6	3	33.3	6	66.7	9

N_1_ = Yes, N_2_ = No, N_1+2_ = Sum of the examinable cases

### Porotic hyperostosis

**Fig 3:**
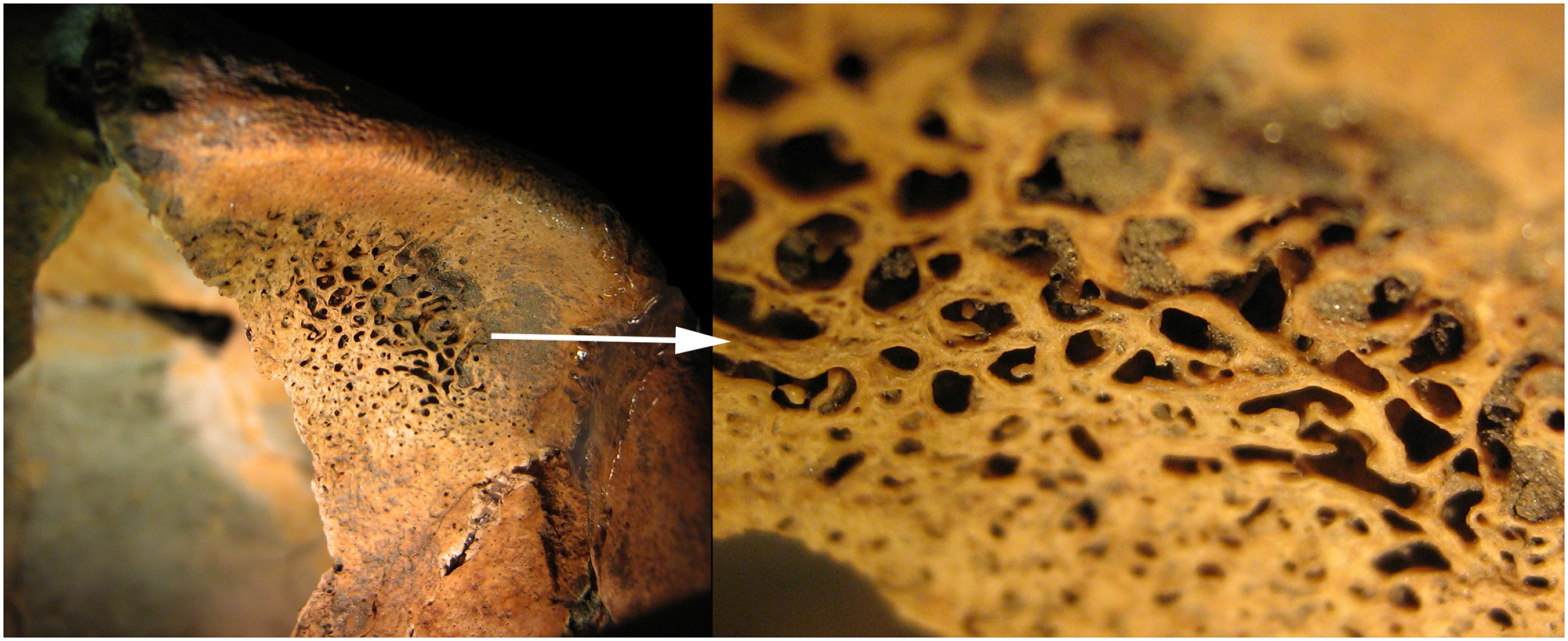
Porotic hyperostosis on the orbital roof, feature 250 S6.

Porotic hyperostosis, suggesting susceptibility for infections, a weakened immune system or haematological disorders [35], was infrequent in this population (Tables 5 and 6). Among the examinable 10 subadults we detected porotic hyperostosis in two individuals. In the 23 adult skulls it was visible in four individuals. Most of them occurred in the orbital roof (*cribra orbitalia*) (Fig 3) and only once on the occipital bone (*cribra cranii*) (Table 5). This alteration affected mainly females, which corresponds to the paleopathological literature (Table 6) [55,56].

**Table 5:** The occurence of porotic hyperostosis.

	Bones	0	%	1	%	2	%	3	%	∑
Sub-adults	Left orbit	5	62.5	2	25.0	1	12.5	0	0.0	8
	Right orbit	7	70.0	1	10.0	2	20.0	0	0.0	10
	Left parietal bone	9	90.0	0	0.0	1	10.0	0	0.0	10
	Right parietal bone	9	100.0	0	0.0	0	0.0	0	0.0	9
	Occipital bone	7	100.0	0	0.0	0	0.0	0	0.0	7
Adults	Left orbit	16	72.7	4	18.2	2	9.1	0	0.0	22
	Right orbit	19	86.4	1	4.5	2	9.1	0	0.0	22
	Left parietal bone	23	100.0	0	0.0	0	0.0	0	0.0	23
	Right parietal bone	24	100.0	0	0.0	0	0.0	0	0.0	24
	Occipital bone	22	95.7	1	4.3	0	0.0	0	0.0	23
Total	Left orbit	21	70.0	6	20.0	3	10.0	0	0.0	30
	Right orbit	26	81.2	2	6.3	4	12.5	0	0.0	32
	Left parietal bone	32	97.0	0	0.0	1	3.0	0	0.0	33
	Right parietal bone	33	100.0	0	0.0	0	0.0	0	0.0	33
	Occipital bone	29	96.7	1	3.3	0	0.0	0	0.0	30

0 = not present, 1 = porotic grade, 2 = cribrotic grade, 3 = trabecular grade

**Table 6:** The occurence of porotic hyperostosis according to sex.

	Grade	Males		Females		Sum
		N	%	N	%	N
Left orbit	0	4	57.14	9	75.0	13
	1	1	14.29	3	25.0	4
	2	2	28.57	0	0.0	2
	3	0	0.00	0	0.0	0
			100.0		100.0	
Right orbit	0	4	66.7	11	91.7	15
	1	0	0.0	1	8.3	1
	2	2	33.3	0	0.0	2
	3	0	0.0	0	0.0	0
			100.0		100.0	
Left parietale	0	7	100.0	12	100.0	19
	1	0	0.0	0	0.0	0
	2	0	0.0	0	0.0	0
	3	0	0.0	0	0.0	0
			100.0		100.	
Right parietale	0	7	100.0	13	100.0	20
	1	0	0.0	0	0.0	0
	2	0	0.0	0	0.0	0
	3	0	0.0	0	0.0	0
			100.0		100.0	
Occipital bone	0	6	100.0	12	100.0	18
	1	0	0.0	0	0.0	0
	2	0	0.0	0	0.0	0
	3	0	0.0	0	0.0	
			100.0		100.0	

0 = Not present, 1 = Porotic grade, 2 = Cribrotic grade, 3 = Trabecular grade

### Signs of a specific infectious disease

Lesions were observed on the facial skeletons and postcranial bones of five individuals, that suggest the possible presence of a specific infectious disease.

#### Feature 257 S20: 18-22-year-old male

**Fig 4:**
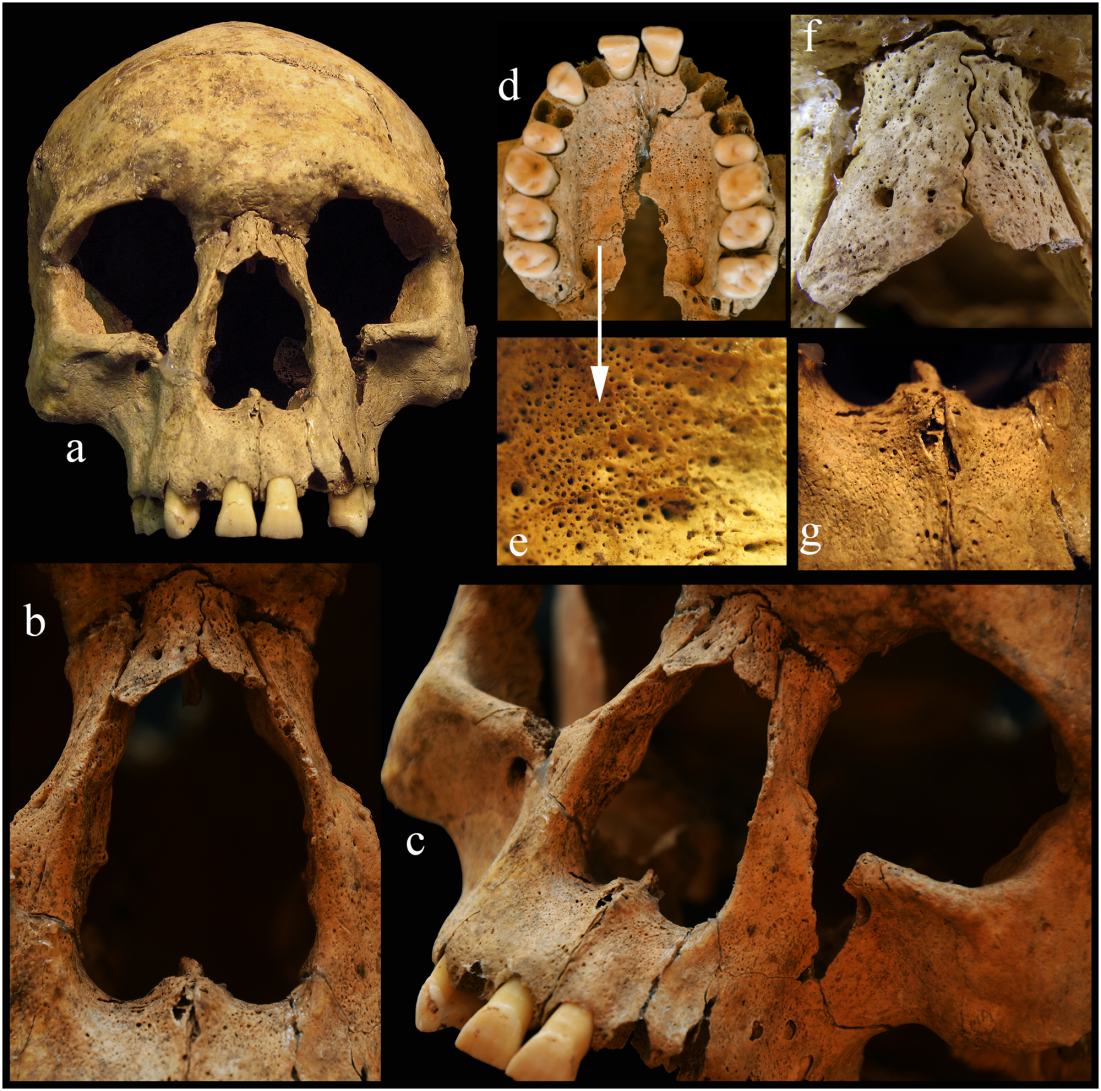
a-c: Rhinomaxillary syndrome on the skull, feature 257 S20. d-e: The hard palate is porotic. f-g: The nasal bones and the anterior nasal spine have atrophied with serious inflammation.

**Fig 5:**
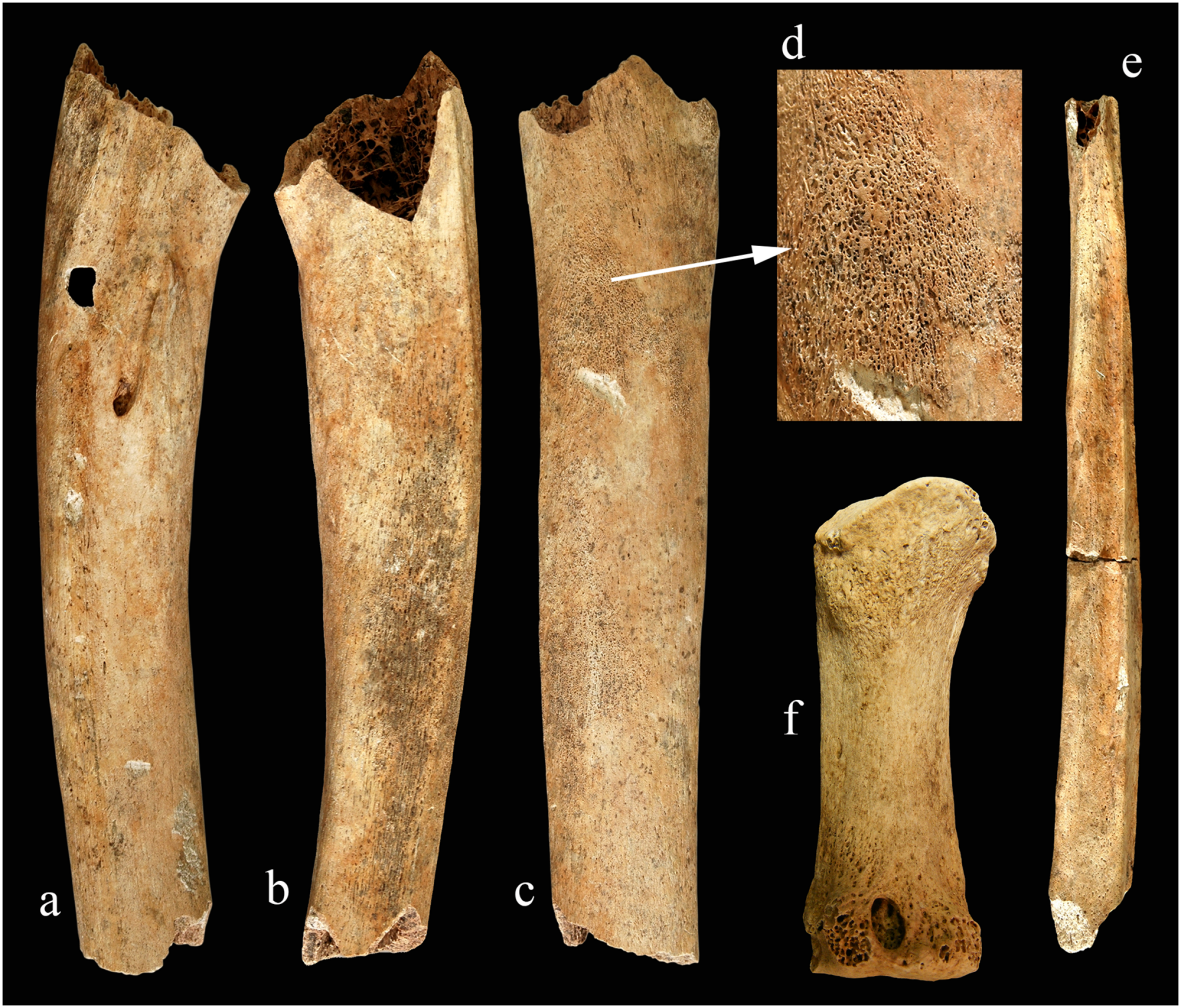
Changes on the extremities (a-d: right tibia e: and fibula; f: right 1st metacarpal bone), feature 257 S20.

A part of the basal and the occipital regions of the skull are missing. The postcranial bones are fragmentary and partially missing. Most of the characteristic alterations are located mainly on the facial skeleton and on the lower limb bones (Figs 4a–4g and 5a–5f).

- smooth remodelling of the margin of the nasal aperture (Fig 4a–4c);
- complete resorption of the anterior nasal spine (Fig 4a–4c and 4g) and the vomer;
- serious inflammation, periostitis and resorption on the nasal bones (Fig 4f);
- porosity on the frontal process, on the infratemporal region of the maxilla, on the left maxillary sinus and on the hard palate (Fig 4d and 4e);
- porosity on the cortical layer of the occipital bone, near the lambdoidal suture;
- pit formation on the cortical layer of the mandible below the incisors;
- porotic vertebral bodies;
- periostitis and hypervascularisation on the ribs;
- periostitis on the right tibia and fibula with abnormal morphology of these bones (Fig 5a–5e);
- periostitis on the diaphyseal part of both humeri (mainly on the left deltoid tuberosity), on the medial surface of both radii, on both ulnae distal end, on both femurs (from the distal of the lesser trochanter) and on the plantar surface of the right sided first os metacarpal with a deep cavity (Fig 5f);
- *cribra orbitalia* on the left orbital roof;
- two healed antemortem trauma in the frontal bone (see above the detailed description of the injuries–S1 Fig).

In a further four cases (feature 263 S25 –S10 Fig; feature 263 S36 –S11 Fig; feature 263 S29 –S12 Fig; feature 263 S39 –S13 Fig) we observed some bone lesions which possibly suggest the presence of leprosy infection but the diagnosis is very uncertain. We provide the detailed description of the pathological lesions of these individuals in the S2 Text.

#### *Mycobacterium leprae* and *Mycobacterium tuberculosis* aDNA analysis

The five macroscopically suspected leprosy cases were subjected to aDNA analysis of the human bones as described above, using three separate sets of extractions. Traditional PCR analysis was performed on the first two sets of extractions, with three primer pairs targeting an outer 129bp and inner 99bp sequence, or a one-stage 111bp sequence. Real-Time PCR analysis was used in the third set of extractions using RLEP primers and probe for an 80bp target and RepLep primers and probe for a 66bp target [16]. Unfortunately, no *M*. *leprae* ancient DNA was detected in the bone samples examined, although positive DNA extraction controls were successful. Neither was *M*. *tuberculosis* detected in the batch 1 extractions.

#### Differential diagnosis

In leprosy, destruction of the nasal bones, nasal septum and conchae, and the hard palate are the common complications on the skull [35,57]. Møller-Christensen [58,59] observed that in archaeological human remains infected by leprosy, the atrophy of the anterior nasal spine and the premaxillary alveolar process with or without loss of the upper incisors, the rounding and widening of the nasal aperture, destruction of the nasal septum and of the hard palate can be found. Neurotrophic alterations of the hands and feet are common bone lesions observed in advanced leprosy. Neurologic problems of the foot can lead to the destruction of the foot, with serious bone loss, severe disfigurement and loss of biomechanical function. Periostitis on the bones of the lower extremities (tibiae, fibulae and the bones of the feet) can be caused by chronic infection of the feet [35].

Some of the skeletal anomalies typical of leprosy also occur in other diseases, such as fungal, treponemal, and oral infections, maxillary sinusitis and leishmaniasis [35], also skeletal tuberculosis, brucellosis and neoplastic disease (metastatic carcinoma).

Fungal infections often cause lytic lesions in the bones. For example, cryptococcosis usually affects the cranial and occasionally other bones, however new bone formation is uncommon. Mucormycosis destroys the nasal cavity, the maxillary sinus and the hard palate, but causing only unilateral perforations. Maduromycosis is distributed in tropical and subtropical regions, while sporotrichosis occurs in humid areas [60]. In all fungal infections the bony changes are unilateral, there is only little destruction and no marginal repair or remodeling [61]. In our cases the lesions are not unilateral and some cases demonstrate a subperiosteal bone reaction. Maduromycosis is excluded due to its geographic range and sporotrichosis is excluded due to its link with high humidity.

In a treponemal disease, such as syphilis, one of the most characteristic lesions is the classical “sabre” shin of the tibiae. Gummatous lesions and a striated nodule on the tibial shaft and a rebuilt trabecular system of the tibia can also occur. Alterations in the skull (mainly in the cranial vault) are frequent and are produced by a combination of destruction and healing accompanied by osteosclerosis (*caries sicca*). Remodeling of the nasal aperture (including loss of the nasal spine) can occur [35,62,63]. The Abony individuals demonstrate no evidence of these treponemal diagnostic criteria such as: *saber tibiae*, gummatous lesions and a striated nodule on the tibial shaft, newly built spongiosa in the tibia, widespread periostitis in the axial and appendicular skeleton, irregular, thick long bones, cavitating lesions on the cranium or *caries sicca* in the cranial vault.

Oral infections and rhinomaxillary sinusitis can cause inflammatory changes to the rhinomaxillary region, and can lead to antemortem tooth loss and destruction of the alveolar part of the maxilla or the mandible [61]. Although in leprosy antemortem tooth loss is frequent, in the Abony cases this phenomenon was not observed. In the case of feature 257 S20, the alveolar process of the anterior part of the maxilla had started to atrophy without any serious inflammation.

Mucocutaneous leishmaniasis can also cause destructive alterations on the bones of the face, with particularly periosteal reactions around the anterior nasal spine and nasal antrum. These affect the mucosal tissues resulting in severe disfigurement of the face. However, there are no lesions on the long bones [35,61]. The alterations on the face are not known to cause destruction of the piriform aperture and the nasal spine that are important diagnostic criteria for leprosy. In the cases presented in this study the atrophy of the nasal spine and the piriform aperture is present.

Bone lesions in skeletal tuberculosis are located most frequently in the spine, tarsals, metatarsals, carpals and metacarpals, knee, hip and elbow joints. Bony manifestations of tuberculosis are well-known and include lytic lesions, vertebral collapse, gibbus, reactive bone formation, etc. The skull (cranial base, cranial vault, and the face) is a very rare area of involvement and occurs mainly in childhood. The cranial vault is the most frequent location of cranial tuberculosis, and the base of the skull is rarely involved. Among the facial bones, the maxilla, especially at the junction with the zygoma, is affected and in the zygomatic arch there may be abscess involvement of temporal bone and the mastoid process, together with a periosteal reaction. The nasal cavity may be affected in a secondary reaction [35]. The skeletal remains at the Abony site are of adults and they do not demonstrate the classic manifestations or localisations consistent with tuberculosis (lesions on spine, joints and other postcranial bones).

Brucellosis is an infectious disease in which the domestication of animals plays an important role. Skeletal involvement of this disease is rare. The most common skeletal lesion is in the spine or sacroiliac joint, long bones are relatively involved. In the spine, brucellosis does not result in collapse of the vertebral bodies or angular kyphosis. Brucellosis causes destructive lesions of the anterior vertebral bodies with reactive projections, and multifocal cavitating abscesses with perforation of the intervertebral disk [35]. The vertebrae and other postcranial bones of our cases show no such multifocal lytic lesions.

In neoplastic diseases (metastatic tumors) the bones are commonly involved. There are two types of bony alterations: osteolytic and osteoblastic. Osteolytic formations may be manifested in the skull and on the facial bones. Carcinoma of the nasal cavity causes destruction of the hard palate and the inner portion of the maxilla, whereas carcinoma of the ethmoid may destroy the frontal bone and the orbital wall [35,64]. However, in all cases the complete reactive bone is absent. These lesions, the absence of the reactive bone and the strictly localized region of the destruction differ from the paleopathology of leprosy cases.

**Fig 6:**
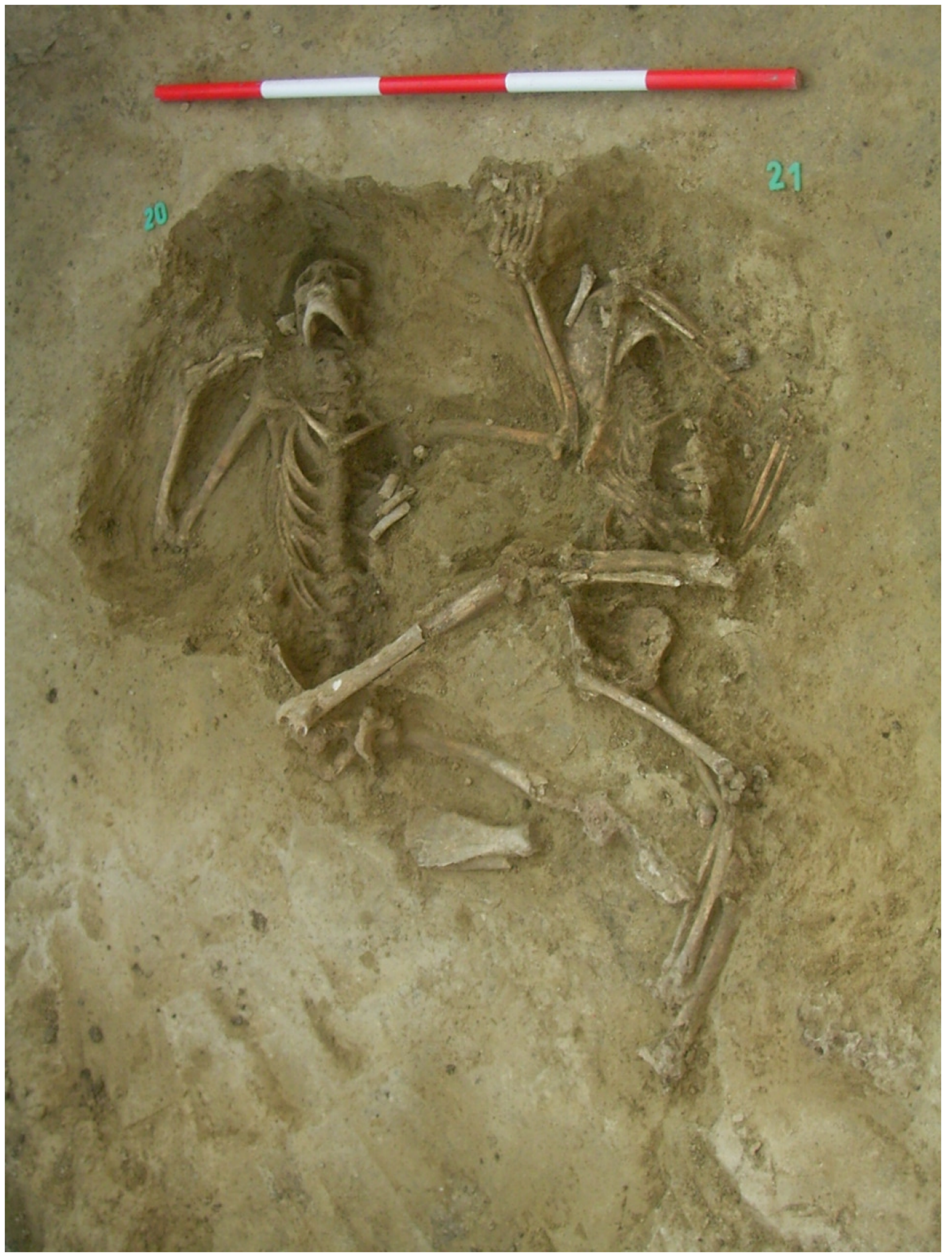
The young male laid on his back, who was thrown into the pit; feature 257 S20.

Based on the above-mentioned diagnostic criteria, the young male from feature 257 S20 shows strong evidence for the bony manifestation of advanced leprosy (Figs 5 and 6).

Based on the Modified Istanbul Protocol [65] this case shows the typical signs of leprosy. In the mass grave the male is laid on his back and he was thrown into the pit with no sign of any burial rite (Fig 6).

In a further two cases (feature 263 S36 –S11 Fig; feature 263 S39 –S13 Fig) there is a suspicion of a leprosy infection. Although other infections cannot be excluded, using the Modified Istanbul Protocol, the paleopathology is highly consistent with leprosy [65]. On the skull of feature 263 S39 the inflammation in the premaxillary area may be caused by an oral infection originating from three abscesses localized to the roots of the upper incisors (S13b Fig).

## Discussion

The archaeological circumstances of the Abony site are significantly different from the pits and settlements that are known from the early, Protoboleráz phase of the Late Copper Age Carpathian Basin [6,7,13]. Based on these archaeological data the ritual origin of the features cannot be excluded. It was proved first, that some pits were thoroughly prepared, before throwing or burying the bodies. Secondly, all of the pits had the same shape. Moreover, the filling in the pits consisted of alternating layers of black humus and sterile loess layers, which suggests a repeated use of the pits. Finally in other pits, there was a posthole-like hollow at the bottom. In many cases systematically placed animal skulls, partial animal skeletons and intact vessels were found in these hollows [5]. The age distribution of the Abony population gave unexpected results when compared with the published paleodemographical data from the investigated region and period (Table 7).

**Table 7:** Percent proportion of fetuses and neonates in the population of Abony and other prehistoric series from the Carpathian Basin.

Archaeological site	Age (A.), archaeological culture (c.) / group (g.)	Number of individuals	Number of fetuses and neonates	Fetuses and neonates (%)
Tiszapolgár-Basatanya* [66]	Copper A., Tiszapolgár c.	59	0	0.0
Tiszapolgár-Basatanya* [66]	Copper A., Bodrogkeresztúr c.	87	0	0.0
Hejőkürt-Lidl Logisztikai Központ [67]	Neolithic, Eastern Linear Pottery c. (ALPC/ELPC)	38	0	0.0
Balatonszárszó-Kis-erdei dűlő [68]	Neolithic, Linear Pottery c. (LPC)	43	0	0.0
Nitra-H. Krškany [69]	Neolithic, LPC	73	1	1.4
Aszód-Papi földek [70]	Neolithic, Lengyel c.	197	5	2.5
Mezőcsát, Bodrogkeresztúr, Tiszapolgár-Csőszhalom, Tiszapolgár, Tiszapolgár-Basatanya [71]	Copper A., Bodrogkeresztúr c., Baden c.	71	2	2.8
Budakalász-Luppa csárda [72]	Copper A., Baden c.	406	13	3.2
Polgár-Ferenci hát [73]	Neolithic, ALPC	120	4	3.3
Hajdúnánás-Eszlári út [73]	Neolithic, ALPC	47	2	4.3
Mórágy B.1 [74]	Neolithic, Lengyeli c.	82	5	6.1
Balatonőszöd-Temetői dűlő [75,76]	Copper A., Baden c.	26	2	7.7
Alsónémedi [25]	Copper A., Baden c.	44	4	9.1
Mezőkövesd-Mocsolyás [73]	Neolithic, Szatmár g.	28	3	10.7
Jánoshida-Berek [77]	Bronze A., Tumulus c.	165	22	13.3
Abony-Turjányos dűlő (present study)	Copper A., Protoboleráz horizon	48	16	33.3

*anthropological results of János Nemeskéri were cited by I. B. Kutzián [66].

The ratio of the newborns and fetuses (0–1 years old children, 33,3%) is extremely high. We compared this result with the data from the anthropological literature (Table 7) involving those series (Abony, Alsónémedi, Budakalász, Jánoshida, Mórágy, Polgár-Ferenci hát) where the number of the individuals in this age group was more than three.

Statistical analysis including Chi-square and Fisher’s exact test were undertaken to determine the differences in the ratio of 0–1 years old in these populations. The ratio of the newborns and fetuses demonstrated a significant difference between the compared populations (*χ*^*2*^ = 51.949 DF = 5 two tailed p<0.001; Fisher’s exact test: 41.659, two tailed p value < 0.001). In a pairwise comparison with the Boneferroni correction, the proportion of the 0–1 years old age goup from the Abony population differed significantly from Polgár-Ferenci hát and Budakalász (p < 0.001). There were no significant differences (p < 0.001) with the Mórágy (p = 0.015) Alsónémedi (p = 0.285) and Jánoshida (p = 0.180) populations.

We present a high number of ante-mortem healed trauma cases in the Abony population (S1 Text). The skull injuries are typical of the use of blunt or round-edged objects, with only a small number apparently caused by wedges, triangular shaped weapons or tools. The depth of the wounds is minimal and only the external surface and the diploë of the skull are affected, suggesting that these were caused by minimal force. Most of them have healed well, with only a few cases showing signs of inflammation. These skull wounds indicate interpersonal violence among the members of the community, rather than warfare. The observed fractures of the long bones are typical of the lifestyle or may also be the result of interpersonal violence.

The observed peri-mortem trauma may have occurred as a result of intra- not inter-group violence. In these cases the ritual activity cannot also be excluded. In previous studies more cases of peri-mortem trauma have been published from the Neolithic and Copper Age [78,79], but based on our recent knowledge there is no other example in the prehistorical Europe for human bone being used as a weapon.

The detailed paleopathological observations suggest that the Copper Age population of Abony suffered from one or more serious infectious diseases, including leprosy. Among the palaeopathologies that suggest infection are endocranial lesions, especially on the occipital bone and periostitis on the long bones, which have occurred in a high proportion of bones in children.

Earlier studies have stated that the fetuses can become infected with *M*. *leprae* during pregnancy [80,81]. However, cord blood IgA is significantly increased in babies of mothers with lepromatous leprosy (LL) and IgA anti-*M*. *leprae* antibodies are present in 30% of cord sera of babies of mothers with active LL [82]. These children have a lower birth weight than the average and suffer from fetal distress [55,83,84]. Moreover, children born to mothers with leprosy are at increased risk of developing leprosy. The time it takes to develop varies according to different authors, some stating that leprosy develops by early childhood, around 2 to 5 years old or by puberty [82,85,86]. There is limited evidence of congenital or peri-natal leprosy, with the suggestion that this is often self-healing [82]. However one case of an infant aged 3–4 months with intra-cranial leprosy pathology has been described in Byzantine Turkey, suggesting there was active infection [86].

In the advanced stage of leprosy typical bone alterations (so-called *facies leprosa*, rhinomaxillary syndrome, lesions on the bones of the hand and feet, and especially penciling of the phalanges) can be found [58,87]. In the Abony material such typical bone alterations were recognized in one individual (Figs 4 and 5). In the case of further four individuals the observed lesions suggest the possibility of leprosy infection. Although the results of the aDNA analysis did not confirm the presence of *M*. *leprae*, this is not proof of absence. DNA is a relatively fragile molecule and its persistence depends on the local environmental conditions of the skeletal remains [45,88,89]. Initial biomolecular analyses were performed on the Abony samples to detect the possible presence of *M*. *leprae* specific mycolic acids and proteins [90,91], but the data require confirmation.

A combination of technologies can help elucidate the origin of bacterial pathogens, their distribution and spread in human populations. This information is relevant today as leprosy is still a global problem. Descriptions of bones with typical leprosy palaeopathology from the pre-historical period have been known for decades.

Written descriptions of alterations consistent with leprosy can be found in ancient Chinese scrolls that refer back to 1122 BC [92]. *M*. *leprae* is an obligate pathogen with no known environmental reservoir. Therefore, its spread around the world is linked to human migrations. According to one opinion, leprosy originated in India and the soldiers of Alexander the Great brought it into the ancient Mediterranean World after the 4^th^ century BC [33,93]. Another suggestion is that the disease evolved in Africa [94], which is supported by *M*. *leprae* genomic data [95,96]. Monot et al. [95] presumed that all contemporary strains of leprosy can be originated to a single clone and the analysis of single nucleotide polymorphisms (SNPs) demonstrated possible origin and dispersal routes through time. They suggested that the ancestral strain of *M*. *leprae* could be either SNP1 or, more likely, SNP2. According to their opinion it is possible that *M*. *leprae* (SNP 2) appeared first in East Africa or in the Near East, with SNP 1 evolving from SNP 2, and then spreading to Asia. SNP 3 also appears to have evolved from SNP2 and was spread to Europe and the Middle East via human migrations associated with the ancient Silk Road. The Americas acquired leprosy via population movements from Europe to the Americas. Later SNP 4 arose in West Africa and thence to the Caribbean via the slave trade. Their alternative suggestion was that SNP 1 appeared in Asia, SNP 2 developed from SNP 1, SNP 3 evolved from SNP 2 and SNP 4 originated from SNP 3. In a later study, Monot et al. [96] found new evidence for 16 SNP subtypes. They suggested that there were two main routes for the spread of leprosy, a northern and a southern route. The northern route brought SNP 3K from the Mediterranean and Turkey, along the Silk Road, to Iran, China and East Asia. The southern route brought SNP 1 from India to Indonesia and the Philippines.

The possible appearance of leprosy in the Copper Age in the Carpathian Basin could be due to the human migrations that originated from the Near East. Earlier physical anthropological papers [97–99] and archaeogenetical papers [100–102] give strong support for this migration route from the Near East to the Balkan penninsula and to the Carpathian Basin in the Early Neolithic (approx. 6000 BC).

The paleopathological evidence demonstrates that leprosy was present in the Old World in prehistory. A possible leprosy case was described from the Anatolian Bronze Age (2700–2300 BC) [103] and from Balatal, Rajastan in India from the late Indus civilisation (2500–1700 BC) [61]. Another ancient putative leprosy case in Western Europe, a child with slight rhinomaxillary changes [104], was found in Dryburn Bridge, Scotland (2300–2000 BC). In Iron Age Italy at Casalecchio di Reno, Bologna, Mariotti and colleagues [105] described a skeleton (4^th^-3^rd^ century BC) with typical morphological bone signs of leprosy. Leprosy cases also have been found in Uzbekistan, dating to the 1^st^-4^th^ century AD [106], in 1^st^ century Israel [107] and at the Dakhleh Oasis in Roman Egypt, dated to the early-mid 4^th^ century AD [108]. Further cases of lepromatous leprosy have been described from mummified remains in early Christian Nubia [109] and the Byzantine period [86,110].

In Hungary the earliest written source about leprosy dates to 1082 AD[111] and Pálfi described the first leprosy case recognized by skeletal remains [112]. Subsequently an ancient *M*. *leprae* case from the Avar Period, dating to the 6-8^th^ century AD, was described [113]. In recent years, many more leprosy cases have been diagnosed and, by using molecular analysis, have provided unequivocal evidence of this disease in Medieval Hungary. Most of these leprosy cases dated from the 7^th^-9^th^ and 10^th^-11^th^ centuries AD [16]. In Western Europe, the peak of morbidity was in the 12^th^-13^th^ century. In the late Middle Ages there were about 19,000 leprosaria (dwellings to segregate individuals with leprosy) throughout the continent, which indicates both the increasing numbers of those infected and the fear of contagion [33,34]. Leprosy was endemic in Europe until the 16^th^ century, but today cases from the indigenous population have almost completely disappeared [114,115].

## Conclusions

Based on the appearance and frequency of healed ante- and peri-mortem trauma we can conclude that inter-personal (intra-group) violence was characteristic in the Abony Late Copper Age population. However other traces of violence were observed on the bones that appear not to have been caused by warfare or inter-group violence. Mass graves are usually associated with large epidemics. In spite of this, there were no signs of any epidemic that may have caused the bony alterations that were observed. There were layers between the human bodies which means these individuals had not died at the same time. The appearance of peri-mortem trauma is also evidence that supports the absence of an epidemic.

Based on recent results there is no way to determine the potential connections between the cases of diagnosed leprosy and the special burial circumstances.

The morphological diagnosed leprosy case from the Abony site, dated to the Late Copper Age (3780–3650 cal BC), is more than one thousand years older than the earliest known case from the Anatolian region [103]. This result significantly modifies our recent knowledge about the time and geographic spread of this specific infectious disease. These observations may stimulate other researchers to re-analyse the bioarchaeological human collections for similar findings that can provide more ancient osteological evidences for the presence of leprosy in the Old World.

## Supporting information

S1 Text Ante and Peri-mortem trauma. (DOCX)

S2 Text Description of the pathological lesions of feature 263 S25, feature 263 S36, feature 263 S29, feature 263 S39. (DOCX)

S1 Table Primers and probes to detect *M*. *leprae* aDNA. (DOCX)

S2 Table The analysed human remains. (DOCX)

S3 Table The occurrence of ante-mortem trauma among adults. (DOCX)

S4 Table The occurrence of ante-mortem trauma according to sex. (DOCX)

S1 Video 3D animation of the pits/ feature 251. The animations were made by Zsolt Réti (Institute of Archaeology, Research Centre for the Humanities, Hungarian Academy of Sciences).
(ZIP)

S2 Video 3D animation of the pits/ feature 257. The animations were made by Zsolt Réti (Institute of Archaeology, Research Centre for the Humanities, Hungarian Academy of Sciences).
(RAR)

S1 Fig Healed depression fractures on the frontal bone, feature 257 S20. (TIF)

S2 Fig Healed triangular and an elongated shaped depression fracture on the left parietal bone (a) and an elongated lesion due to a blow on the left side of the frontal bone (b), feature 257 S11. (TIF)

S3 Fig Healed wound with slight inflammation on the left parietal bone, feature 257 S12. (TIF)

S4 Fig Porosity and inflammation on the external surface of the skull vault and wound with sign of inflammation on the left parietal bone, feature 263 S10. (TIF)

S5 Fig Healed fracture on both ulnae, feature 263 S29. (TIF)

S6 Fig Female stabbed with an animal bone (horn), feature 257 S12. (JPG)

S7 Fig A female individual stabbed with a human fibula, feature 263 S22. (TIF)

S8 Fig Female stabbed with human fibula, feature 263 S22. The fibula penetrated into the body of the 12^th^ thoracic vertebra.
(TIF)

S9 Fig This image is shows that a large stone was thrown into the feature 263, after the deposition of individuals, S25 and S28,which fractured the humerus of S25 (a-b) and the tibia of S28 (c-e). (TIF)

S10 Fig a-c: Rounded margins and horizontal vein grooves of the piriform aperture. d: Periostitis on the right tibia may be caused by trauma; feature 263 S25.
(TIF)

S11 Fig a-c: Possible rhinomaxillary syndrome. d: Healed periosteal lesion on the tibia. e-f: Periostitis and cavity formation on the left heel bone; feature 263 S36.
(TIF)

S12 Fig Atrophied lateral margin with semicircular shape of the piriform aperture, feature 263 S29. (TIF)

S13 Fig a-c: Atrophied lateral margin and inflammation of the piriform aperture and three abscesses at the roots of the upper incisors. d: ossification in the left external auditory pore; feature 263 S39.
(TIF)
