# Supplementary material for: Possible cases of leprosy from the Late Copper Age (3780-3650 cal BC) in Hungary
Source: PLoS One. 2017 Oct 12;12(10):e0185966. doi: 10.1371/journal.pone.0185966 (PMC5638319; doi:10.1371/journal.pone.0185966)
Supplement: S1 Text — (DOCX) [file pone.0185966.s001.docx]

**Supplementary text 1**

**Ante mortem trauma**

We analysed the possible presence of ante mortem trauma on the human bones of 48 individuals (25 subadults and 23 adults). There were no detectable signs of trauma on the long bones or skulls of infants. From the 23 adult skulls available for investigation, traces of trauma were visible in four cases (three males and one female) (S3-S4 Tables). An irregular shaped healed depression fracture, approximately 10 mm wide, was found on the frontal bone of a 18-22 year old male (feature 257 S20) (S1 Fig). The lesion was located 12 mm from the coronal suture on the right side of the frontal bone. The injury affected only the external cortical layer and the diploë. The surface of the lesion is pitted which indicates inflammation due to the healing process. Beside it, another healed trauma was detected about 9 mm from the meeting point of the temporal line and the coronal suture. More specifically, it is located partially on the coronal suture and partially on the parietal bone. The shape of the injury is oval and the maximal diameter is 5 mm. It is assumed that this was caused by blunt force, which also affected the external cortical layers and the diplöe.

In case of a 35-45 year old male (feature 257 S11), approximately 3-4 mm far from the left parietal bone, all of the external cortical, the diploë and the internal cortical were damaged, although the inner part is only slightly visible (S2 Fig). The blunt trauma of the parietal bone caused a 2-3 mm depression into the cranial cavity, and here branching of the meningeal grooves is slightly altered. The injury may have been caused by a sharp-edged, triangular shaped stone or a metal (copper) tool. The healing process was accompanied by serious inflammation, shown by the porotic surface. On the left side of the frontal bone, 8 mm above the temporal line, a ca. 13 mm long and 3-4 mm wide injury can be seen on the external cortical surface and the diploë. Presumably it was caused by a low velocity blow.

On the left parietal bone of a 25-35 year old female (feature 257 S12), ca. 16 mm below the frontal eminence, a roughly oval-shaped lesion is visible, 5 mm in diameter, suggesting blunt force trauma (S3 Fig). The lesion was healed with slight inflammation, the cortical layer is porotic and irregular, while the diploë is not affected.

In a 35-40 year old male (feature 263 S10), there is a triangular shaped lesion, with a slightly porotic surface on the left parietal bone, 10 mm from the coronal suture and approximately 40 mm from the superior temporal line (S4 Fig). The diameter of the wound is 3-4 mm. The injury may have been caused by a stone axe, or possibly a metal (copper) tool.

The 262 analysed long bones belonged to 23 adult skeletons (10 males and 13 females). In 15 cases the ribs (7 males and 8 females), and in five cases (1 male and 4 females) the sternum was investigated. Fractures or cracking were observed on eight long bones of four individuals (three males and one female).

On an undeterminable-sided fibula fragment from a 30-40 year old male (feature 263 S7) a healed fracture with callus formation and serious angulation was detected.

The right fibula of a 35-45 year old male (feature 263 S25) was fractured in the mid-part of the diaphysis and healed with slight angulation. Periosteal apposition had developed on the lateral surface of the right tibia, presumably due to the same traumatic event.

The distal diaphyseal ends of both ulnae of a 35-45 year old female (feature 263 S29) were broken (S5 Fig). The left ulna had healed with slight callus formation and angulation. Due to this injury rim formation and signs of inflammation are visible on the wrist joint. The right ulna had healed with serious callus formation and inflammation. The surface of the left radius (approximately at the height of the same sided ulna fracture) is very uneven with no sign of fracture or crack. The right radius was unexaminable. The injury of the right ulna is typically produced when an individual tries to protect their head or neck with raised forearms ("parry”-fracture).

In the case of a 35-45 year old probable male (263 S30), the distal diaphyseal end of the left ulna shows a healed fracture with angulation, inflammation and callus formation. Beside it, in the mid-part of the right fibula, periostitis with severe inflammation was detected, which had also slightly affected the right tibia. These alterations could be caused by a minor trauma and resultant cracking of the bone.

In the case of a 35-40 year old female (feature 263 S39), multiple (six) rib fractures were observed. One of the left hand phalanxes of a 30-40 year old female (feature 263 S28) demonstrated a healed fracture with minimal callus formation.

**Peri-mortem trauma**

During the excavation and the physical anthropological analysis, unambiguous traces of peri-mortem physical violence were found in three cases.

Between the 6^th^ and 7^th^ right ribs of a 25-35 year old adult female (feature 257 S12) an animal bone (horn) had penetrated through the ribs (S6 Fig). There were no traces of any fracture or other lesion that may have been caused by the animal bone. It could only have reached the soft tissue under the rib cage.

An earlier fractured human fibula was stabbed into the body of the 12^th^ thoracic vertebra (263 S22) of a 25-30-year-old female (S7 Fig). The bone firstly intruded into the abdomen, which could cause significant soft tissue injury, but was stopped by the lowest thoracic vertebra. On the CT scan and 3D reconstruction of this vertebra, it can be clearly seen that the fibula penetrated several more millimetres deep into the vertebral body (S8 Fig).

If these two females were alive when these inter-personal violences occured, these events must have significantly contributed to their deaths.

In feature 263 two skeletons were laid on the top of each other. After their deposition a large stone had been thrown on them, which broke the humerus of the adult-mature male (S25) lying on the top and the tibia of the adult female (S28) lying underneath (S9 Fig). Based on the direction and the morphology of the fracture lines, these injuries were very likely to have happened close to their deaths.
