# Supplementary material for: Possible cases of leprosy from the Late Copper Age (3780-3650 cal BC) in Hungary
Source: PLoS One. 2017 Oct 12;12(10):e0185966. doi: 10.1371/journal.pone.0185966 (PMC5638319; doi:10.1371/journal.pone.0185966)
Supplement: S2 Text — (DOCX) [file pone.0185966.s002.docx]

**Supplementary Text 2. Description of the pathological lesions of feature 263 S25, feature 263 S36, feature 263 S29, feature 263 S39.**

**Feature 263 S25: 35-45-year-old male**

The facial skeleton, the skull base and the occipital part of the skull are fragmentary and partially missing. In spite of the fragmentary state of the face the most of the alterations listed below can be seen (S10 Fig).

- - - rounded lateral and inferior margins of the piriform aperture in semicircular shape and horizontal vein grooves in the lower regions of the piriform aperture (S10a-c Fig);
    - slight inflammatory changes in the nasal cavity (S10c Fig);
    - inflammation in the hard palate;
    - slight enlargement of the left maxillary frontal process;
    - porotic surface on the occipital bone near the lambdoid suture;
    - inflammation in the sacroiliac joint;
    - porosity in the bodies of the vertebrae;
    - osteoarthrosis in the shoulder-, elbow-, knee-, ankle- and the all vertebral joints;
    - healed fracture in the right fibula with periostitis on the middle diaphyseal part of the right tibia (probable connected to the trauma) (S10d Fig).

**Feature 263 S36: 40-45-year-old female**

The skull base and the occipital part are fragmentary and partially missing. Most of the alterations are occurred on the facial skeleton and on the long bones (S11 Fig).

- - - Widened inferior and lateral margins of the piriform aperture (S11a-c Fig);
    - inflammation on the parietal bones near the sagittal and lambdoid suturae;
    - thickened frontal and parietal bones on tha calvaria near the “bregma” point;
    - periostitis on the diaphyseal part of both tibiae (S11b Fig), distal epiphyseal ends of both fibulae and on the lateral surface of the calcanei (S11e-f Fig);
    - cavity formation on the calcanei (S11e-f Fig);
    - healed periostitis on the medial surface of the ribs;
    - osteoarthrosis in the lumbar spine, in the right shoulder, in the left wrist and in the left hip joints.

**Feature 263 S29: 35-40-year-old female**

The skull base and the occipital part of the skull are fragmentary and partially missing. Evidence for bone pathology is on the facial skeleton and on the postcranial bones (S12 Fig).

- - - Atrophied lateral margin with semicircle shaped of the piriform aperture (S12 Fig);
    - large abscess in the maxilla at the buccal side of the root of the left upper second incisor (S12 Fig);
    - slight uneven surface on the ribs medially;
    - periostitis on both femora, tibiae and fibulae;
    - inflammation on the left metatarsi and tarsi with marginal osteophyte formation and synostosis;
    - healed fracture in the distal end of the ulna;
    - osteoarthrosis in more joints of the long bones and in the lumbar spine.

**Feature 263 S39: 35-40-year–old female**

The facial skeleton and the occipital part of the skull are fragmentary and partially missing. Alterations are on the skull and on the post-cranial skeleton (S13 Fig).

- - - Atrophied lateral margin of the piriform aperture (S13a-c Fig);
    - inflammatory changes: pit formations and long grooves from the lower margin of nasal aperture to the incisors (S13a-c Fig);
    - puffed up lower part of the nasal bones and of the frontal process of the maxilla (S13a-b Fig);
    - porotic surface with pit formations of the both parietal bones along the sagittal and lambdoid suturae;
    - atrophied (with semicircular form) upper margin of the alveolus of the left upper medial incisor (I1), and resorption on the lower, palatinal margin of the left upper lateral incisor (I2) (S13a-b Fig);
    - porotic postcranial bones;
    - long grooves in the body of all vertebrae;
    - hypervascularisation on both sides of the body of some thoracic and lumbar vertebrae;
    - secondary bony apposition (trace of pleuritis) on the medial surface of the ribs;
    - mild inflammation in the sacroiliac joint;
    - periostitis of the lower extremity;
    - bony apposition in the external acoustic pore, probably caused by traumatic lesion or infection (S13d Fig);
    - osteoarthrosis in articulation of both shoulders, elbows, wrists and hips;
    - periostitis on some ribs with fractures.
