# Supplementary material for: Possible cases of leprosy from the Late Copper Age (3780-3650 cal BC) in Hungary
Source: PLoS One. 2017 Oct 12;12(10):e0185966. doi: 10.1371/journal.pone.0185966 (PMC5638319; doi:10.1371/journal.pone.0185966)
Supplement: S1 Table — (DOCX) [file pone.0185966.s003.docx]

**Supplementary Table 1**

Primers and probes to detect *M. leprae* aDNA

*M.leprae*-specific primers

LP1 RLEP 129 bp ^5'^TGCATGTCATGGCCTTGAGG^3’^

LP2 " ^5'^CACCGATACCAGCGGCAGAA^3’^

LP3 " 99 bp ^5'^TGAGGTGTCGGCGTGGTC^3’^

LP4 " ^5'^CAGAAATGGTGCAAGGGA^3’^

MT2 " 111 bp ^5'^CATTTCTGCCGCTGGTATC^3’^

MT4 " ^5'^ATCATCGATGCACTGTTCAC^3’^

RLEP probe assay:

RlepF: 80 bp ^5’^CGCTGGTATCGGTGTCG^3’^

RLepR: ^5’^ACACGATACTGCTGCACC3’

RLep Probe: FAM-^5’^CTCAGCCAGCAAGCAGGCAT^3’^-BHQ2

RepLep probe assay:

RepLepF: 66 bp ^5’^GACTGTACTTCTTGGCCAGC^3’^

RepLepR: ^5^’GCAAGGTGAGCGTTGTGG^3’^

RepLepProbe FAM-^5’^CATGTCTATCTCCGTACGCAGCTG ^3’^- BHQ1
