# Supplementary material for: Possible cases of leprosy from the Late Copper Age (3780-3650 cal BC) in Hungary
Source: PLoS One. 2017 Oct 12;12(10):e0185966. doi: 10.1371/journal.pone.0185966 (PMC5638319; doi:10.1371/journal.pone.0185966)
Supplement: S2 Table — (DOCX) [file pone.0185966.s004.docx]

**Supplementary Table 2.** The analysed human remains

| Feature no. | Strat. no. | Sex | Age (years) |
| --- | --- | --- | --- |
| 247 | 35 | ? | fetus (9-9.5 lunar month) |
| 249 | 19 | ? | fetus (8.5-9 lunar month) |
| 249 | 31 | ? | fetus (8.5-9 lunar month) |
| 250 | 5 | ? | 5.5-6.5 years |
| 250 | 6 | ♂ | 45-55 years |
| 251 | 16 | ♀ | 30-40 years |
| 251 | 37 | ? | fetus (8.5-9.5 lunar month) |
| 251 | 43 | ? | fetus (9-9.5 lunar month) |
| 251 | 44 | ? | fetus (8.5-9.5 lunar month) |
| 253 | 40 | ? | fetus (9-10 lunar month) |
| 253 | 41 | ? | neonatus (1-3 month) |
| 253 | 47 | ? | fetus (8.5-9.5 lunar month) |
| 253 | 48 | ? | fetus (9-9.5 lunar month) |
| 253 | 49 | ? | fetus (8.5-9.5 lunar month) |
| 253 | 50 | ? | fetus (7.5-8 lunar month) |
| 253 | 51 | ? | fetus (7-7.5 lunar month) |
| 257 | 11 | ♂ | 35-45 years |
| 257 | 12 | ♀ | 25-35 years |
| 257 | 13 | ? | fetus (9.5-10 lunar month) |
| 257 | 14 | ? | 23-39 years |
| 257 | 15 | ♀ | 30-50 years |
| 257 | 17 | ? | 5.5-6.5 years |
| 257 | 18 | ? | 2.5-3.5 years |
| 257 | 20 | ♂ | 18-22 years |
| 257 | 21 | ? | 9-10 years |
| 263 | 4 | ♀ | 40-50 years |
| 263 | 7 | ♂ | 30-40 years |
| 263 | 8 | ♀ | 30-40 years |
| 263 | 9 | ♀ | 45-55 years |
| 263 | 10 | ♂ | 35-40 years |
| 263 | 22 | ♀ | 30-35 years |
| 263 | 23 | ♂? | 25-35 years |
| 263 | 24 | ? | 3.5-4.5 years |
| 263 | 25 | ♂ | 35-45 years |
| 263 | 26 | ♂ | 30-55 years |
| 263 | 27 | ? | 2.5-3.5 years |
| 263 | 28 | ♀ | 30-40 years |
| 263 | 29 | ♀ | 35-45 years |
| 263 | 30 | ♂? | 45-55 years |
| 263 | 34 | ♂ | 45-55 years |
| 263 | 36 | ♀ | 40-45 years |
| 263 | 38 | ? | 1.5-2.5 years |
| 263 | 39 | ♀ | 35-40 years |
| 263 | 42 | ♀ | 35-45 years |
| 263 | 45 | ♀ | 30-35 years |
| 263 | 46 | ? | 8-10 years |
| 263 | 52 | ? | fetus (9-10 lunar month) |
| 263 | 53 | ? | fetus (9-9.5 lunar month) |
