# Supplementary material for: Possible cases of leprosy from the Late Copper Age (3780-3650 cal BC) in Hungary
Source: PLoS One. 2017 Oct 12;12(10):e0185966. doi: 10.1371/journal.pone.0185966 (PMC5638319; doi:10.1371/journal.pone.0185966)
Supplement: S3 Table — (DOCX) [file pone.0185966.s005.docx]

**Supplementary Table 3**. The occurrence of ante-mortem trauma among adults.

|  | Present | % | No present | % | Total |
| --- | --- | --- | --- | --- | --- |
| Skull | 4 | 17.4 | 19 | 82.6 | 23 |
| Left clavicle | 1 | 7.7 | 12 | 92.3 | 13 |
| Right clavicle | 0 | 0.0 | 10 | 100.0 | 10 |
| Sternum | 0 | 0.0 | 5 | 100.0 | 5 |
| Ribs | 1 | 6.7 | 14 | 93.3 | 15 |
| Left humerus | 0 | 0.0 | 16 | 100.0 | 16 |
| Right humerus | 0 | 0.0 | 17 | 100.0 | 17 |
| Left ulna | 3 | 18.7 | 13 | 81.3 | 16 |
| Right ulna | 1 | 6.7 | 14 | 93.3 | 15 |
| Left radius | 0 | 0.0 | 15 | 100.0 | 15 |
| Right radius | 0 | 0.0 | 14 | 100.0 | 14 |
| Left pelvis | 0 | 0.0 | 16 | 100.0 | 16 |
| Right pelvis | 0 | 0.0 | 15 | 100.0 | 15 |
| Left femur | 0 | 0.0 | 21 | 100.0 | 21 |
| Right femur | 0 | 0.0 | 21 | 100.0 | 21 |
| Left tibia | 0 | 0.0 | 19 | 100.0 | 19 |
| Right tibia | 2 | 10.5 | 17 | 89.5 | 19 |
| Left fibula | 1 | 5.9 | 16 | 94.1 | 17 |
| Right fibula | 1 | 5.6 | 17 | 94.4 | 18 |
