# Supplementary material for: Possible cases of leprosy from the Late Copper Age (3780-3650 cal BC) in Hungary
Source: PLoS One. 2017 Oct 12;12(10):e0185966. doi: 10.1371/journal.pone.0185966 (PMC5638319; doi:10.1371/journal.pone.0185966)
Supplement: S4 Table — (DOCX) [file pone.0185966.s006.docx]

**Supplementary Table 4.** The occurrence of ante-mortem trauma according to sex.

| Bones | Males | | | | | Females | | | | |
| --- | --- | --- | --- | --- | --- | --- | --- | --- | --- | --- |
|  | Total | Present | % | Absent | % | Total | Present | % | Absent | % |
| Skull | 8 | 3 | 37.5 | 5 | 62.5 | 13 | 1 | 7.7 | 12 | 92.3 |
| Left clavicle | 3 | 0 | 0.0 | 3 | 100.0 | 8 | 0 | 0.0 | 8 | 100.0 |
| Right clavicle | 3 | 0 | 0.0 | 3 | 100.0 | 7 | 0 | 0.0 | 7 | 100.0 |
| Sternum | 1 | 0 | 0.0 | 0 | 100.0 | 4 | 0 | 0.0 | 4 | 100.0 |
| Ribs | 7 | 0 | 0.0 | 7 | 100.0 | 8 | 1 | 12.5 | 7 | 87.5 |
| Left humerus | 6 | 0 | 0.0 | 6 | 100.0 | 7 | 0 | 0.0 | 7 | 100.0 |
| Right humerus | 6 | 0 | 0.0 | 6 | 100.0 | 8 | 0 | 0.0 | 8 | 100.0 |
| Left ulna | 6 | 1 | 16.7 | 5 | 83.3 | 8 | 1 | 12.5 | 7 | 87.5 |
| Right ulna | 6 | 0 | 0.0 | 6 | 100.0 | 7 | 1 | 14.3 | 6 | 85.7 |
| Left radius | 6 | 0 | 0.0 | 6 | 100.0 | 8 | 0 | 0.0 | 8 | 100.0 |
| Right radius | 6 | 0 | 0.0 | 6 | 100,0 | 6 | 0 | 0.0 | 6 | 100.0 |
| Left pelvis | 6 | 0 | 0.0 | 6 | 100.0 | 10 | 0 | 0.0 | 10 | 100.0 |
| Right pelvis | 6 | 0 | 0.0 | 6 | 100.0 | 9 | 0 | 0.0 | 9 | 100.0 |
| Left femur | 8 | 0 | 0.0 | 8 | 100.0 | 9 | 0 | 0.0 | 9 | 100.0 |
| Right femur | 7 | 0 | 0.0 | 7 | 100.0 | 10 | 0 | 0.0 | 10 | 100.0 |
| Left tibia | 7 | 0 | 0.0 | 7 | 100.0 | 9 | 1 | 11.1 | 8 | 88.9 |
| Right tibia | 7 | 2 | 28.6 | 5 | 71.4 | 8 | 0 | 0.0 | 8 | 100.0 |
| Left fibula | 7 | 1 | 14.3 | 6 | 85.7 | 8 | 0 | 0.0 | 8 | 100.0 |
| Right fibula | 5 | 1 | 20.0 | 4 | 80.0 | 8 | 0 | 0.0 | 8 | 100.0 |
