# Supplementary figures and images for: Possible cases of leprosy from the Late Copper Age (3780-3650 cal BC) in Hungary
Source: PLoS One. 2017 Oct 12;12(10):e0185966. doi: 10.1371/journal.pone.0185966 (PMC5638319; doi:10.1371/journal.pone.0185966)

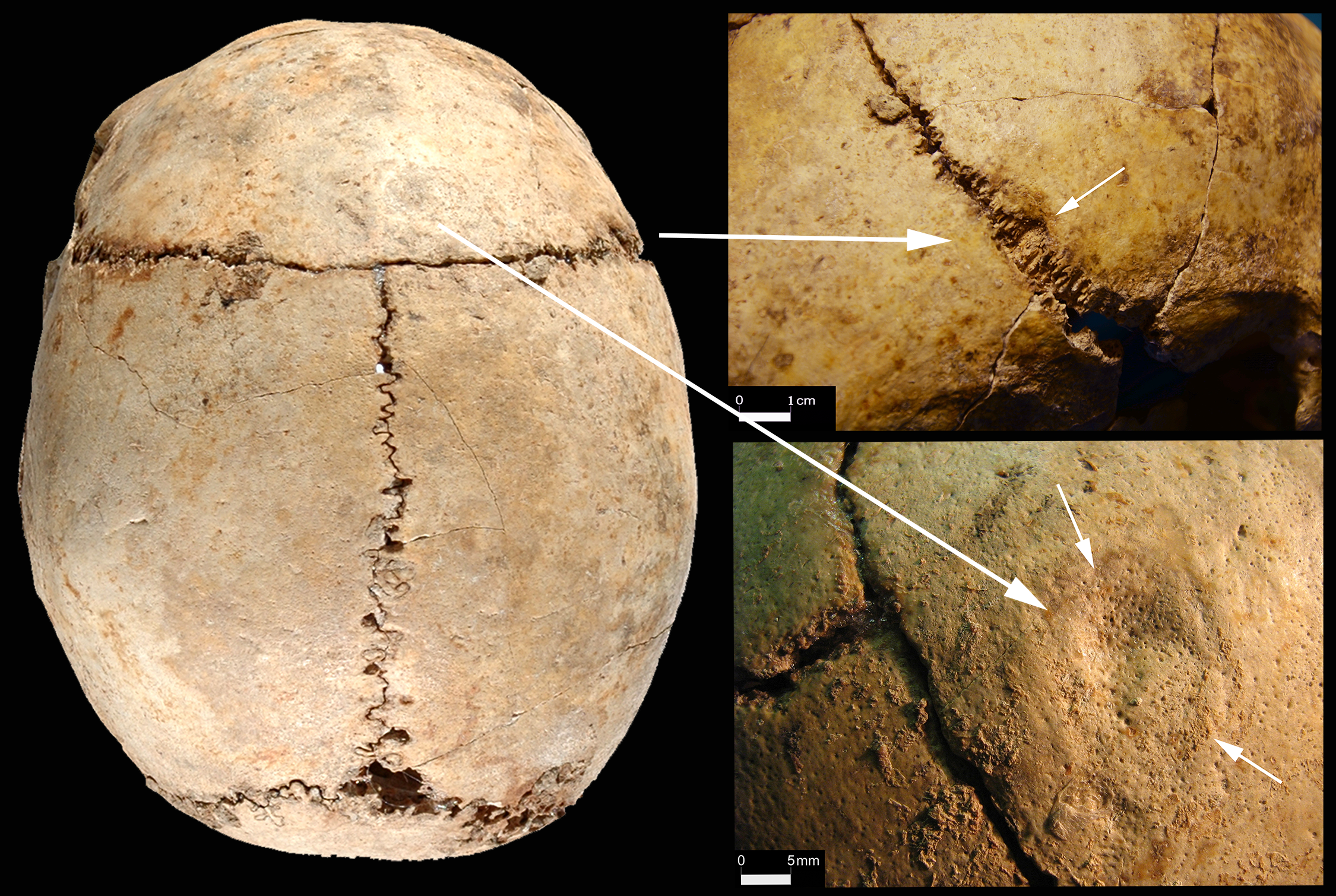

Supplement: S1 Fig — (TIF) [file pone.0185966.s009.tif]

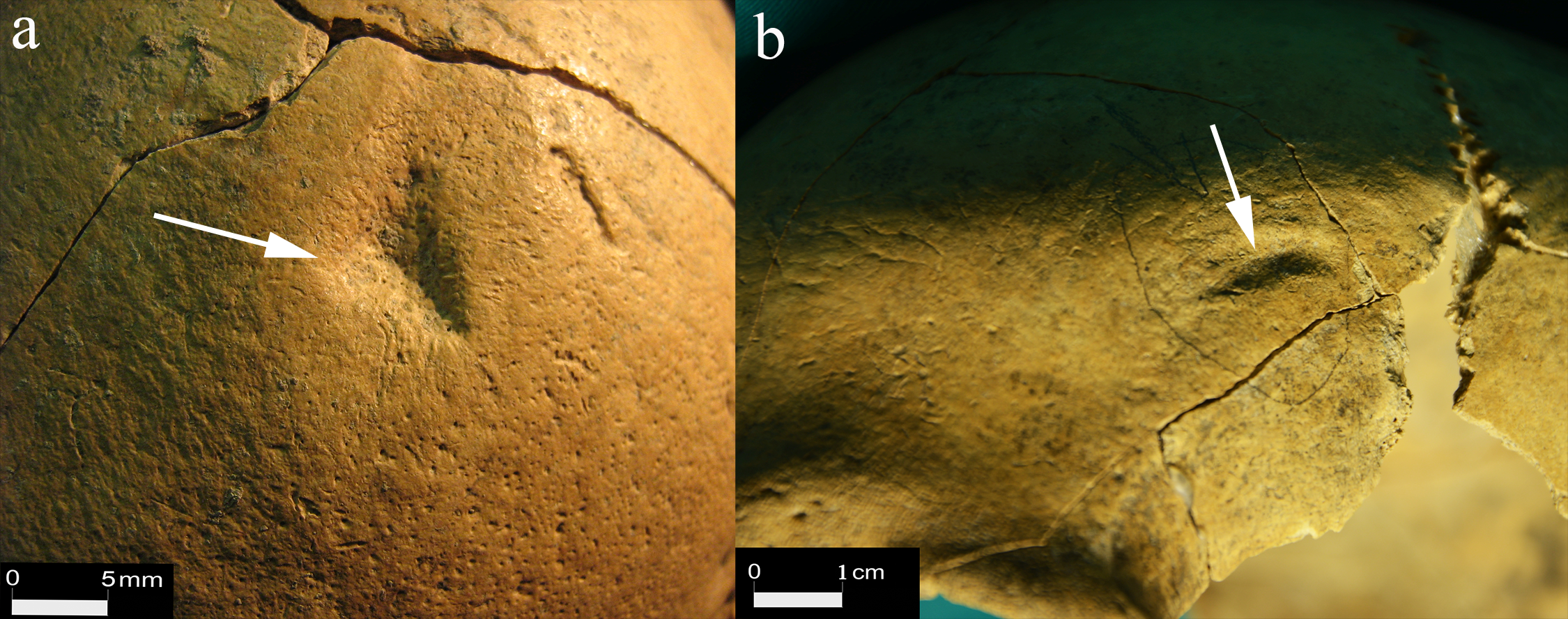

Supplement: S2 Fig — (TIF) [file pone.0185966.s010.tif]

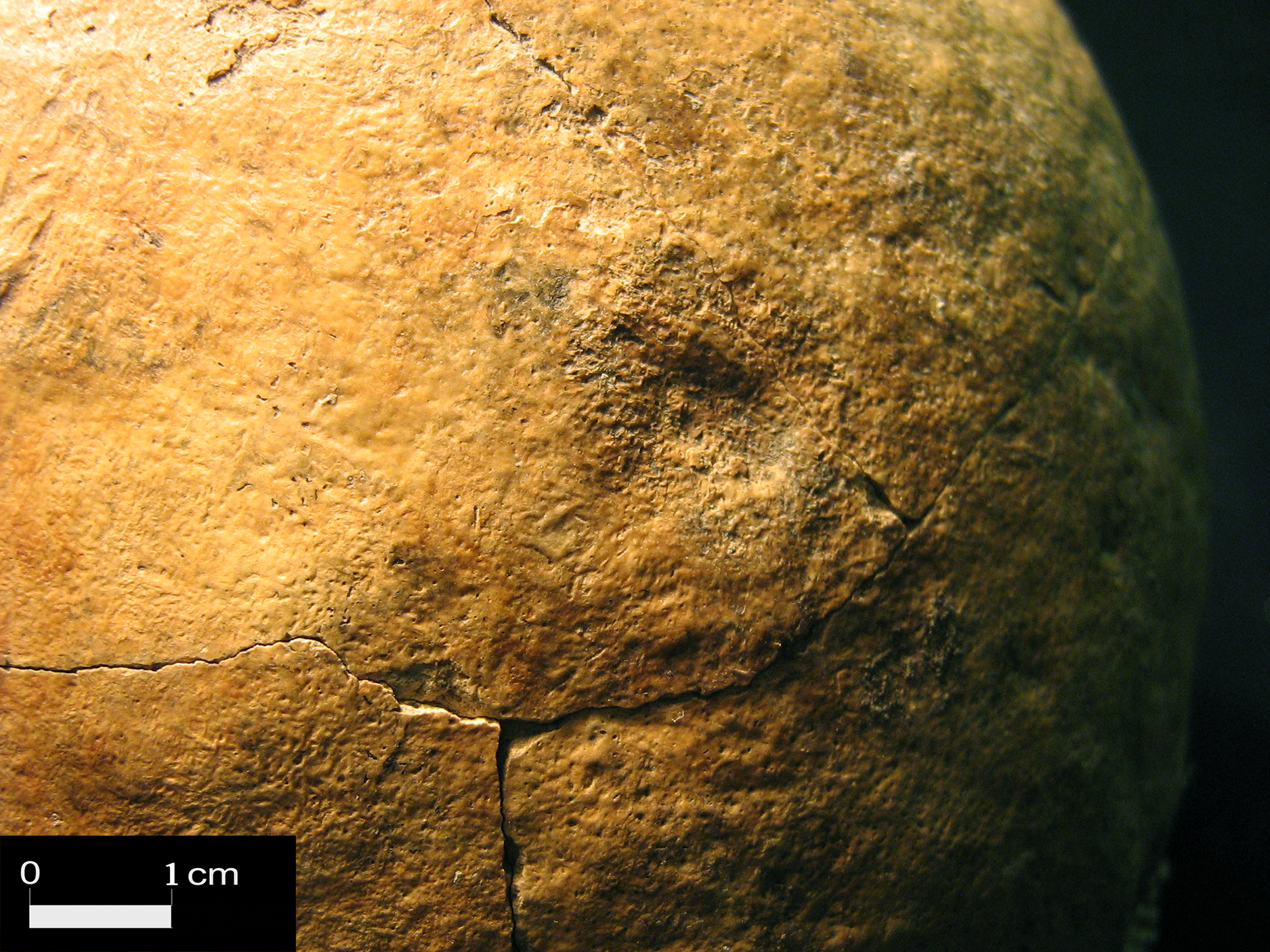

Supplement: S3 Fig — (TIF) [file pone.0185966.s011.tif]

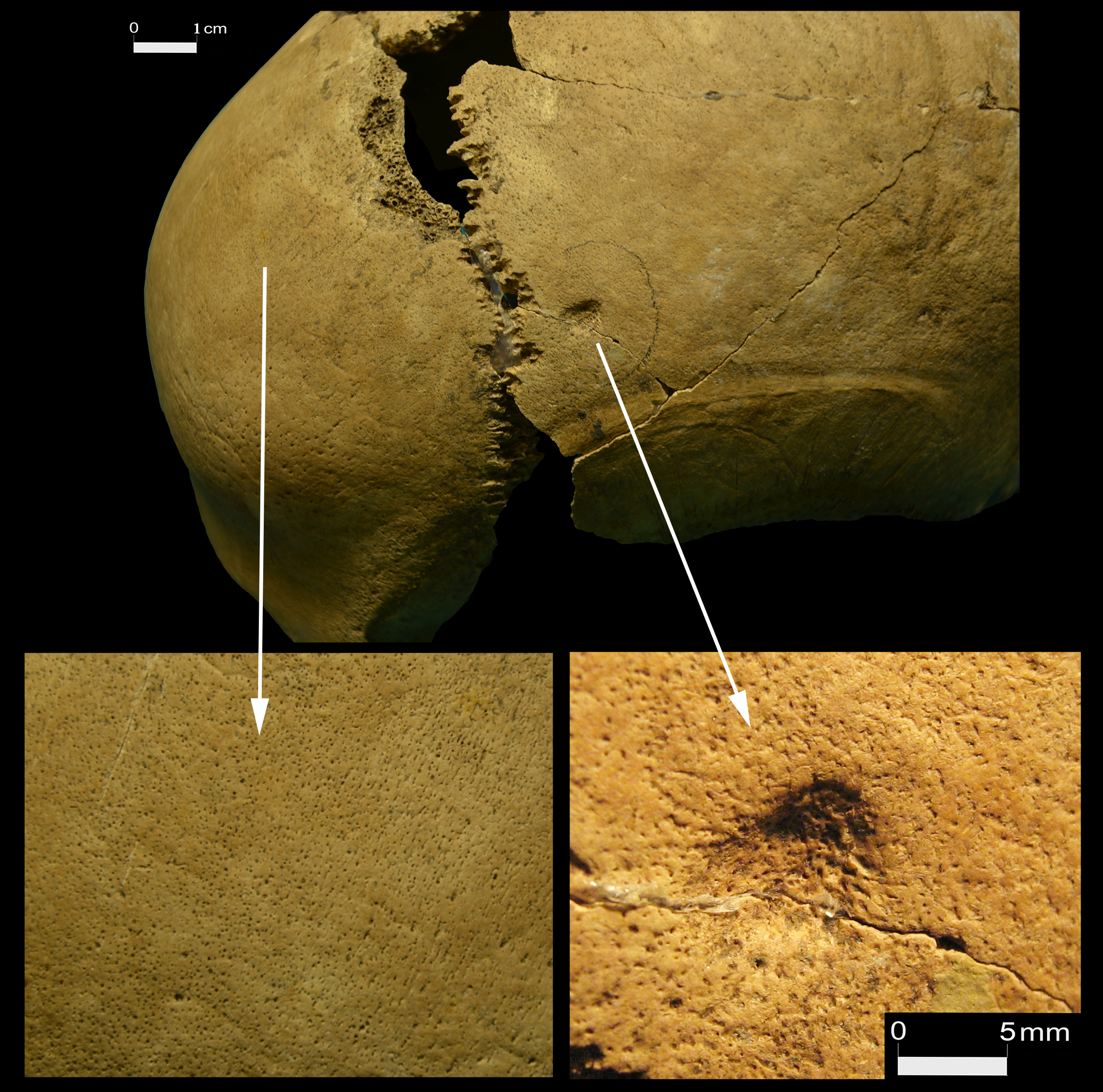

Supplement: S4 Fig — (TIF) [file pone.0185966.s012.tif]

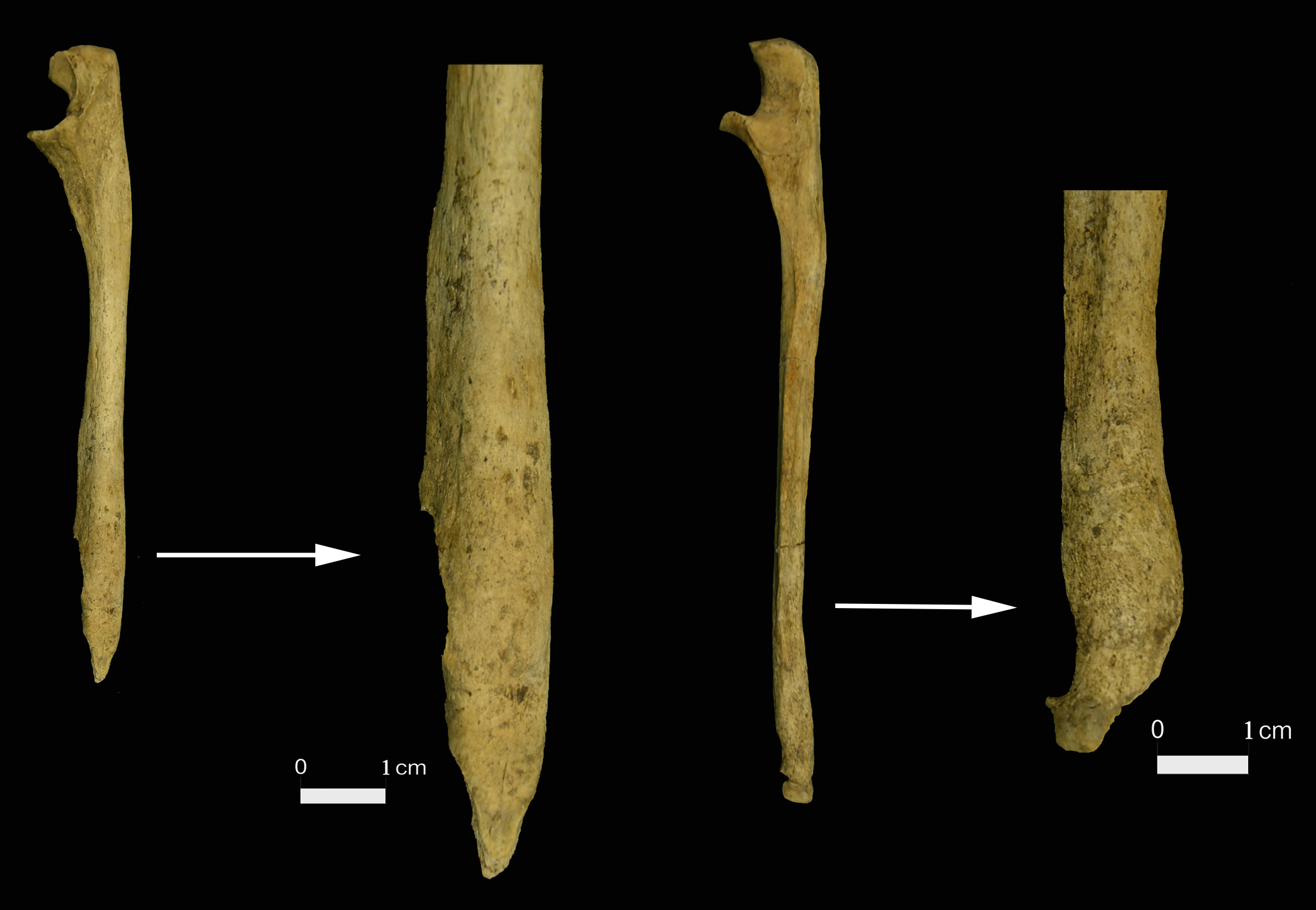

Supplement: S5 Fig — (TIF) [file pone.0185966.s013.tif]

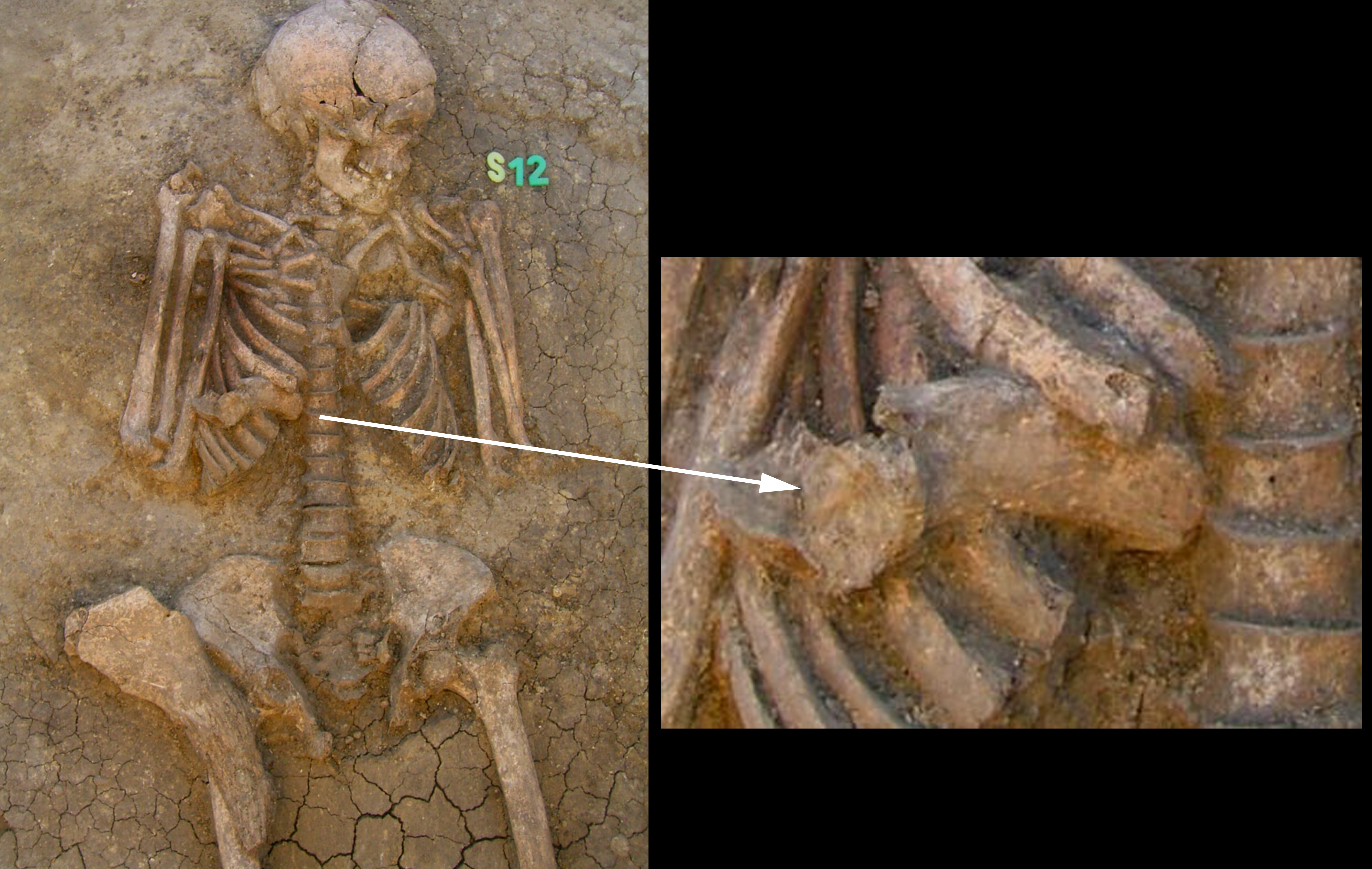

Supplement: S6 Fig — (JPG) [file pone.0185966.s014.jpg]

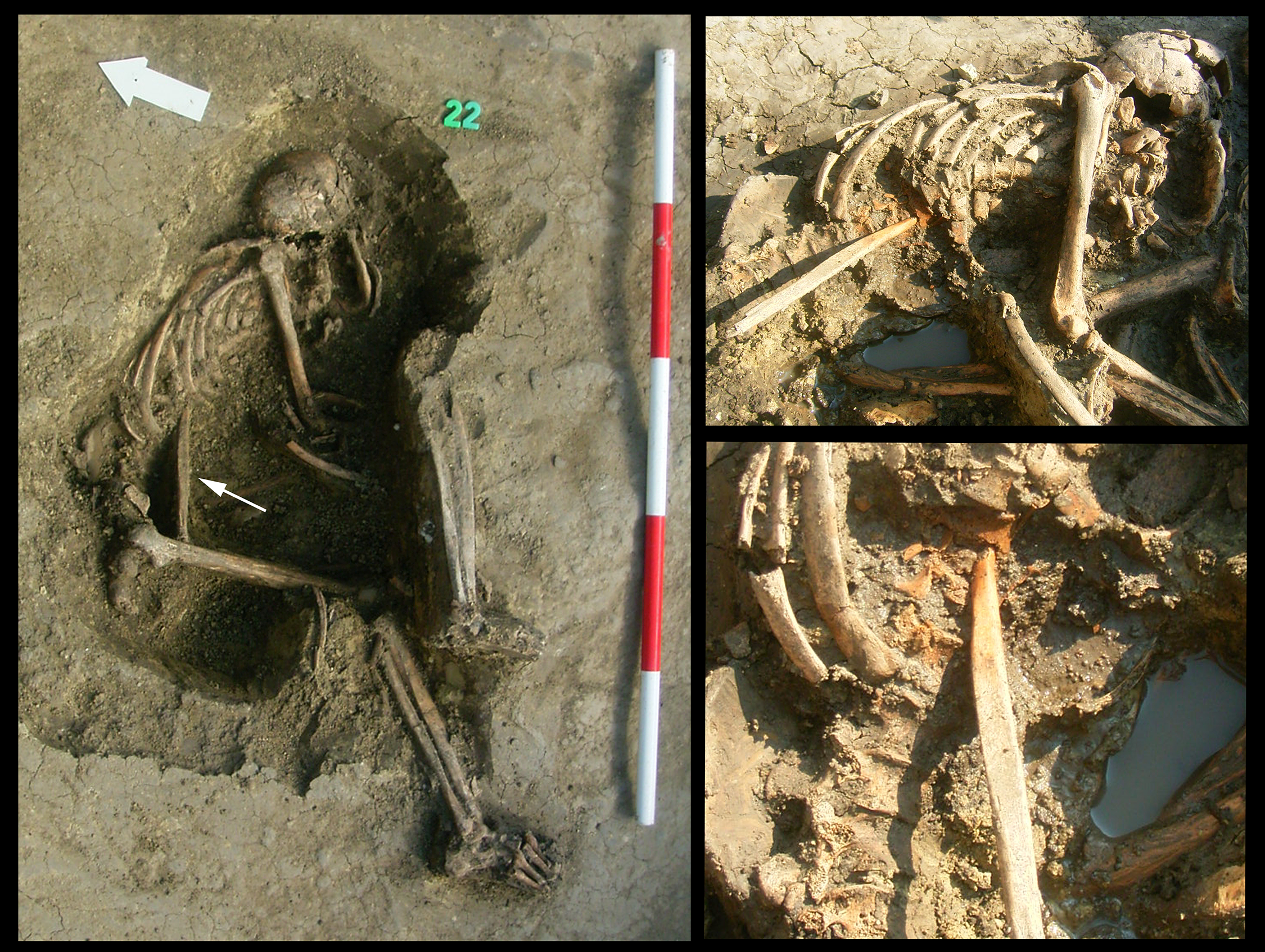

Supplement: S7 Fig — (TIF) [file pone.0185966.s015.tif]

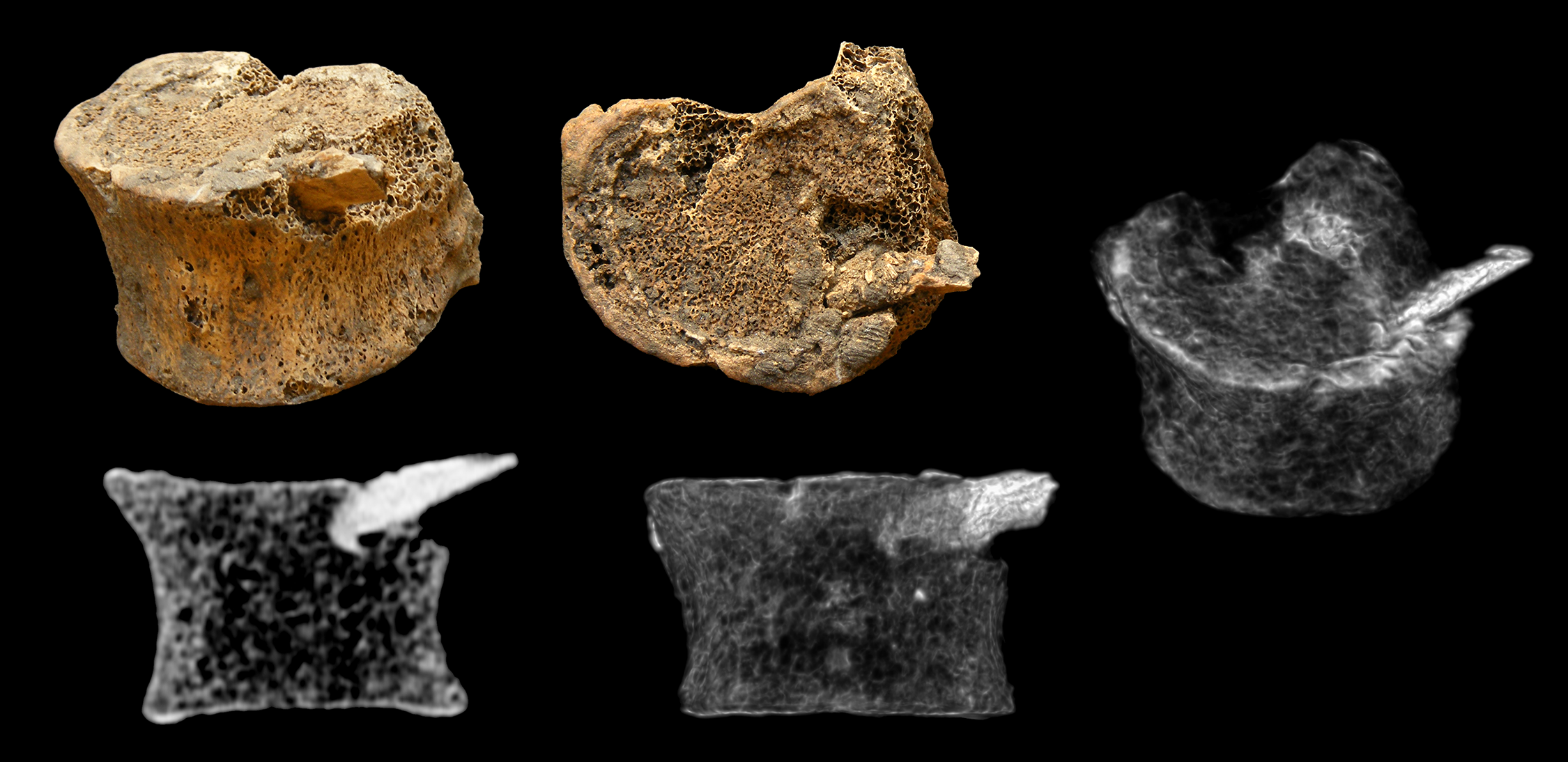

Supplement: S8 Fig — The fibula penetrated into the body of the 12th thoracic vertebra. (TIF) [file pone.0185966.s016.tif]

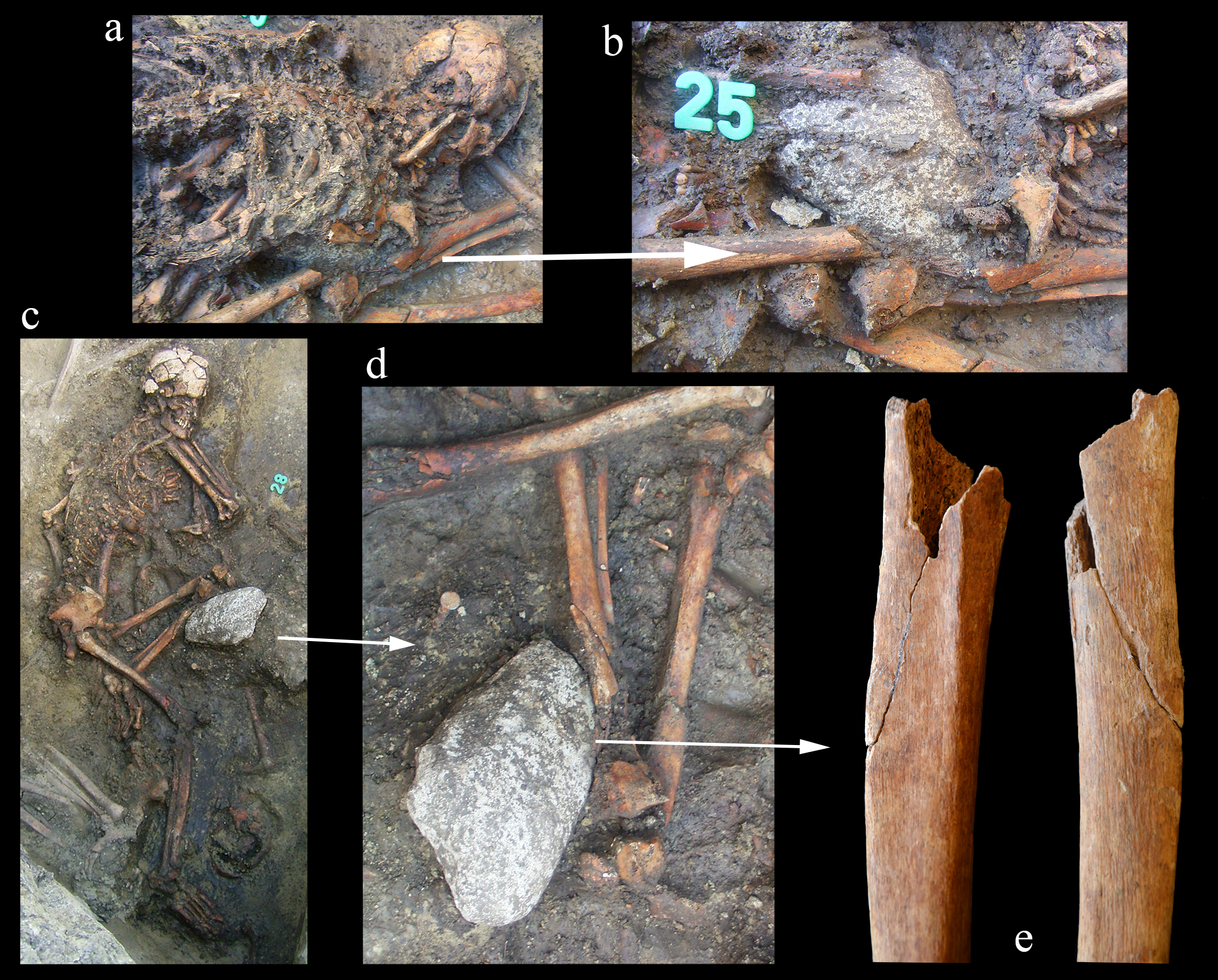

Supplement: S9 Fig — (TIF) [file pone.0185966.s017.tif]

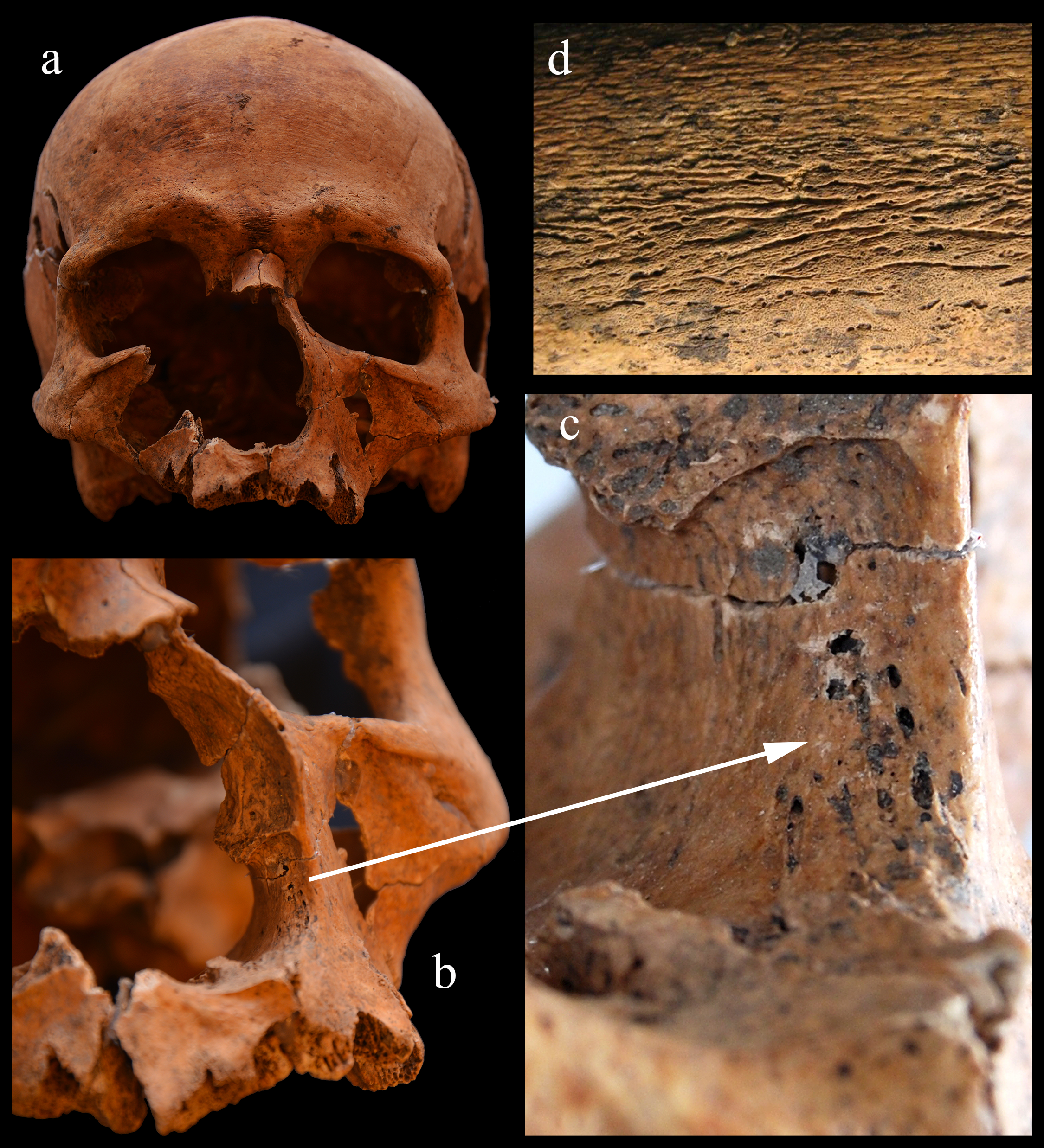

Supplement: S10 Fig — a-c: Rounded margins and horizontal vein grooves of the piriform aperture. d: Periostitis on the right tibia may be caused by trauma; feature 263 S25. (TIF) [file pone.0185966.s018.tif]

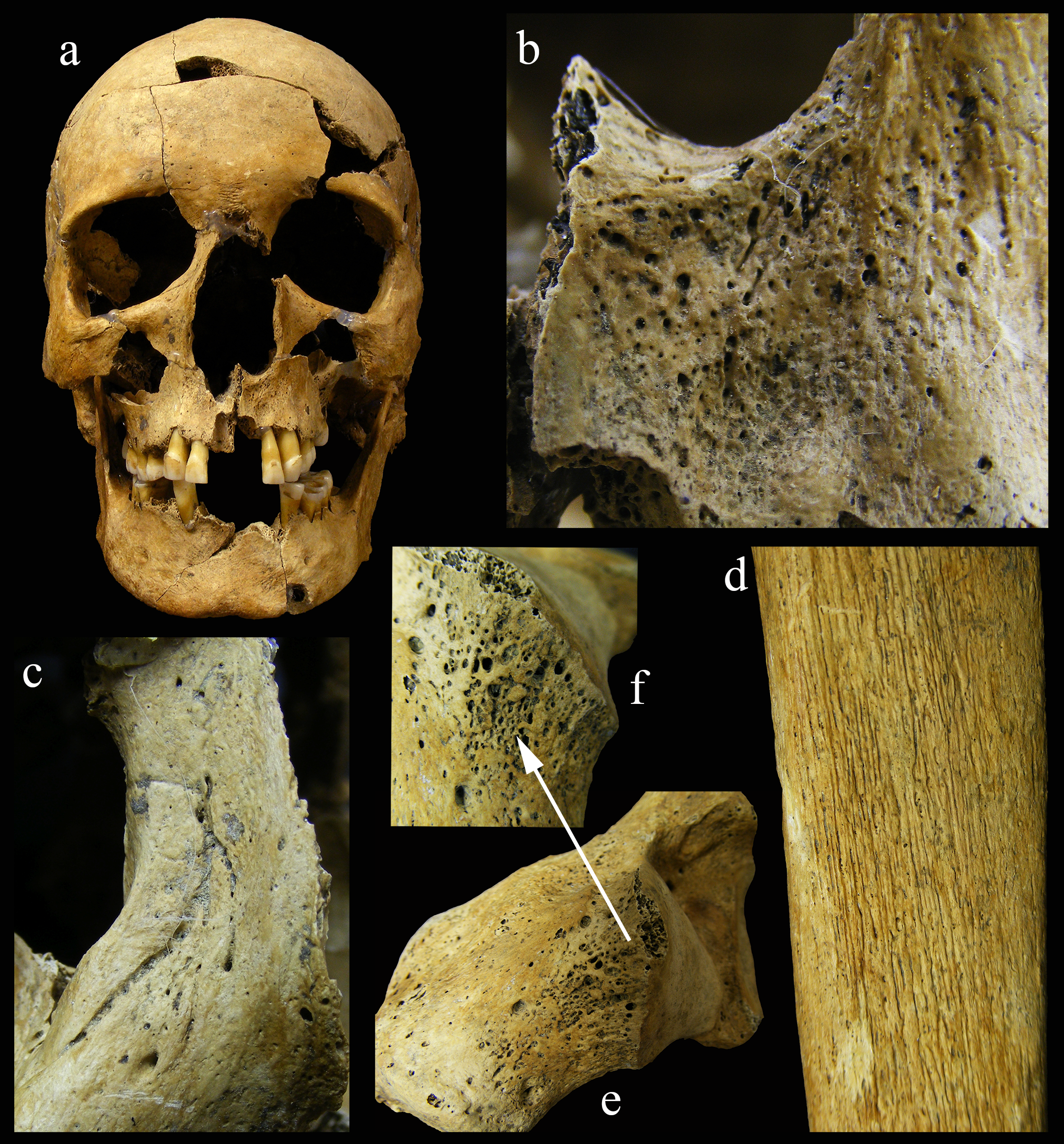

Supplement: S11 Fig — a-c: Possible rhinomaxillary syndrome. d: Healed periosteal lesion on the tibia. e-f: Periostitis and cavity formation on the left heel bone; feature 263 S36. (TIF) [file pone.0185966.s019.tif]

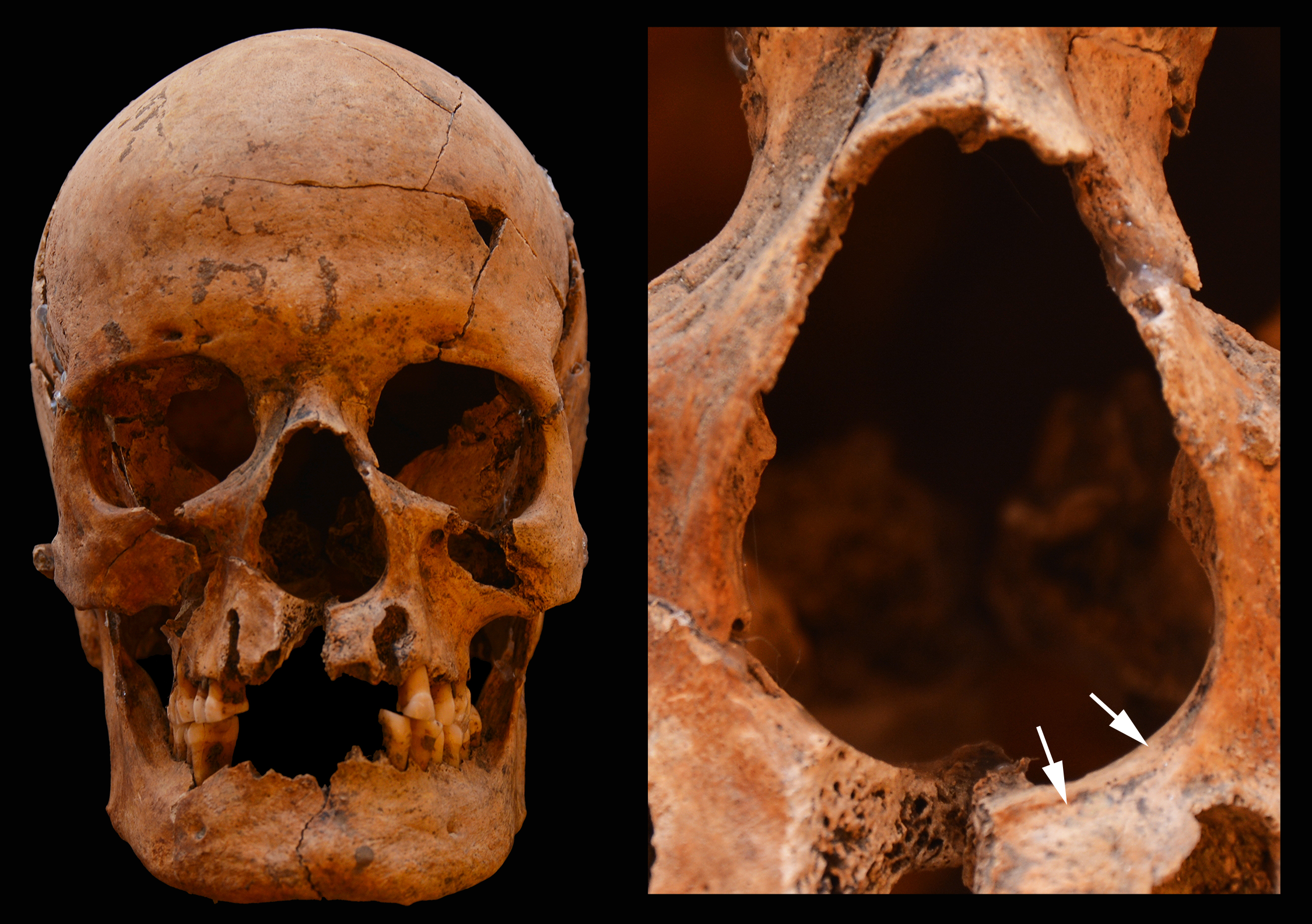

Supplement: S12 Fig — (TIF) [file pone.0185966.s020.tif]

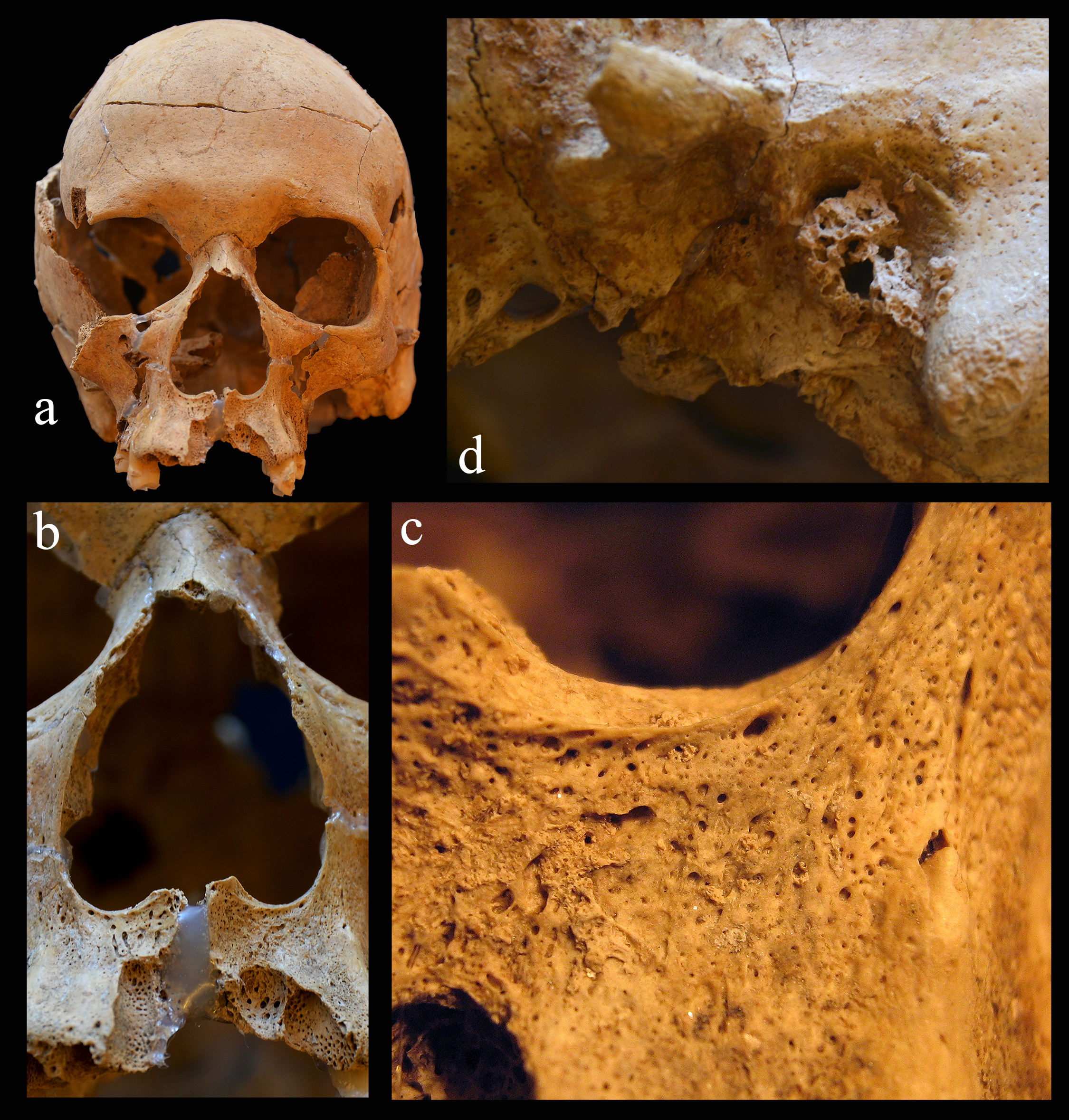

Supplement: S13 Fig — a-c: Atrophied lateral margin and inflammation of the piriform aperture and three abscesses at the roots of the upper incisors. d: ossification in the left external auditory pore; feature 263 S39. (TIF) [file pone.0185966.s021.tif]
